# Organic Electrochemical Transistors Monolithically Integrated with Precise Micro‐Dispensing Enable High‐Performance Biosignals Amplification

**DOI:** 10.1002/advs.202508872

**Published:** 2025-08-21

**Authors:** Roberto Granelli, Virginia M. Demartis, Giulia Frusconi, Zsolt M. Kovács‐Vajna, Fabrizio Torricelli

**Affiliations:** ^1^ Department of Information Engineering University of Brescia via Branze 38 Brescia 25123 Italy

**Keywords:** micro‐dispensing, OECT amplifier, OECT, organic electrochemical transistors

## Abstract

Organic electrochemical transistors (OECTs) are key components in advanced bioelectronics, wearable devices, and neuromorphic biointerfaces. However, to fully unlock their potential and facilitate widespread adoption, the development of high‐performance OECTs and their seamless integration into circuits is essential, yet still limited. In this work, the monolithic integration of OECTs and OECT‐based amplifiers, fabricated entirely on flexible substrates using high‐resolution micro‐dispensing, is reported. This additive method enables precise deposition of conductors, semiconductors, insulators, and electrolytes across a wide viscosity range (10 –10^5^ cP) with micrometer‐scale resolution and femtoliter‐volume control. The resulting transistors achieve a record intrinsic gain of 330 V/V and form the basis for fully printed amplifier circuits with a maximum voltage gain of 77.5 and a supply‐normalized gain of 193.7 V^−1^. These amplifiers demonstrate a gain‐bandwidth product of 1 MHz – the highest reported for fully printed OECTs – and enable real‐time acquisition and amplification of electrooculography (EOG) signals with minimal distortion. This work establishes micro‐dispensing as a scalable and reliable method for manufacturing high‐performance printed bioelectronic circuits, bridging the gap between low‐cost fabrication and the performance requirements of next‐generation bioelectronics.

## Graphical abstract

This research showcases a micro‐dispensing strategy for integrating high‐performance organic electrochemical transistors (OECTs) and amplifiers onto flexible substrates. The method achieves precise material deposition, enabling fully micro‐dispensed OECT amplifiers with a record 1 MHz gain‐bandwidth product. This technology enables real‐time biosignal amplification for advanced bioelectronic applications.

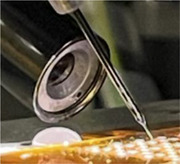

## 1 Introduction

Organic electrochemical transistors (OECTs) have become pivotal components in modern bioelectronics,^[^ ^1^, ^2^ ^]^ offering unique capabilities such as efficient ionic‐electronic coupling,^[^ ^3^, ^4^ ^]^ mechanical flexibility,^[^ ^5^, ^6^ ^]^ and operation at low voltages (<1 V), essential for safe biological interfacing.^[^ ^7^, ^8^ ^]^ Their ability to achieve high transconductance normalized to channel dimensions (up to several of S/cm),^[^ ^9^ ^]^ at sub‐1 V operation makes them ideal candidates for applications ranging from biosensing and health signal acquisition^[^ ^10^, ^11^ ^]^ to neuromorphic^[^ ^12^ ^]^ and wearable electronics.^[^ ^13^ ^]^ Recent advances have extended the application of OECTs to electrophysiological sensors,^[^ ^14^ ^]^ ion detection,^[^ ^15^ ^]^ impedance biosensors,^[^ ^16^, ^17^ ^]^ and large‐area complementary circuits,^[^ ^18^, ^19^ ^]^ highlighting their transformative potential in healthcare and beyond.

Despite these achievements, significant challenges remain in scaling OECT technologies. A major barrier is the reliable fabrication of micro‐scale OECTs and OECT‐based circuits with ultra‐low cost fully‐additive methods, while maintaining high performance. Conventional fabrication processes, primarily based on photolithography, offer high spatial resolution but suffer from critical drawbacks: they require multiple solvent‐intensive steps, are incompatible with many organic materials, and incur high production costs, limiting scalability and environmental sustainability.^[^ ^20^, ^21^, ^22^, ^23^ ^]^ Furthermore, the use of aggressive solvents during photolithography can degrade the performance of active polymer materials, necessitating additional protective layers that complicate device fabrication and increase variability.^[^ ^24^, ^25^ ^]^

Additive manufacturing (AM) techniques – such as inkjet printing, aerosol jet printing, screen printing, and dispensing – have emerged as promising alternatives, offering low‐cost, maskless, and scalable solutions. AM methods eliminate photomasks, reduce material waste, and allow facile integration with flexible and biocompatible substrates, aligning perfectly with the vision of portable, wearable, and implantable electronics. However, state‐of‐the‐art AM techniques also face fundamental limitations. Inkjet and aerosol jet printing, while precise, are constrained to low‐viscosity inks (typically <50−100 cP), limiting material selection and often leading to poor film morphology for organic semiconductors. On the other hand, screen printing and dispensing techniques can handle higher viscosities (>1000 cP) but suffer from lower spatial resolution, insufficient for miniaturized device integration.^[^ ^26^, ^27^ ^]^ These limitations have so far prevented the monolithic integration of OECTs with AM methods. Moreover, most of state‐of‐art printed OECTs rely on hybrid approaches, where only one component (e.g., the electrolyte or the channel) is printed and the electrodes are usually patterned by lithography or shadow masking. Such partial printing strategies, although useful, compromise process simplicity, increase manufacturing costs, and hinder the realization of large‐area, low‐cost OECT‐based bioelectronic systems.^[^ ^28^, ^29^, ^30^, ^31^ ^]^ Therefore, although hybrid fabrication methods have been successfully employed for the realization of organic bioelectronic systems on flexible substrates,^[^ ^32^ ^]^ monolithic fabrication with additive manufacturing techniques can provide several practical and technological advantages, reducing the system complexity, avoiding the need for equipment changes between the various fabrication steps, and lowering the equipment and maintenance costs. Importantly, one of the most critical benefits is the avoidance of substrate alignment between fabrication steps, saving significant time and resulting in improved precision of the alignment between the various material layers. By maintaining consistent deposition and curing conditions within a unified workflow, monolithic methods can enhance feature resolution and pattern fidelity, which are important features for integrating multi‐functional and compact bioelectronic systems.

Moreover, a critical performance bottleneck in OECT‐based electronics for biosensing and biointerfacing is the insufficient signal amplification. Biological signals recorded from the human body often exhibit low amplitudes, from 1 µV to over 10 mV depending on the signal types (e.g., electrooculography, electroencephalography),^[^ ^33^ ^]^ and require high amplifier gains to ensure reliable detection and analysis. While OECTs can deliver high transconductance, integrating them into circuits that can achieve high gain while maintaining low‐voltage operation (<1 V) remains extremely challenging. Most previously reported OECT‐based amplifiers achieve limited gains (<50 V/V) and operate at voltages higher than biologically safe thresholds, significantly restricting their applicability in health monitoring and in‐liquid bioelectronic systems.

Addressing these open challenges – namely, the monolithic, high‐resolution, scalable integration of high‐performance OECTs and circuits with high gain and low‐voltage operation – would represent a major technological leap. It would enable the development of next‐generation bioelectronic platforms that are low‐cost, highly sensitive, and suitable for seamless integration into wearable, implantable, and point‐of‐care systems.

In this work, we overcome these barriers by developing a micro‐dispensing‐based additive manufacturing strategy that enables the monolithic fabrication of OECTs and OECT‐based amplifier circuits directly on flexible substrates. All OECT device and circuit components – including silver gate, gold source and drain electrodes, mixed ionic‐electronic conducting organic channels, and ionic‐electronic insulating layers – are reliably fabricated through the precise deposition of material volumes ranging from 3 to 800 femtoliters (3×10^−15^⁵ to 8×10^−13^ L). This corresponds to a spatial resolution between 2 and 40 micrometers, enabling high‐definition patterning of functional electronic elements on a flexible substrate. Our approach combines the scalability and cost‐effectiveness of additive manufacturing with the performance characteristics of conventional lithographic processes. Overcoming the viscosity limitations of conventional AM methods, the micro‐dispensing approach enables the use of a wide range of materials, achieving micrometer‐scale precision patterning. We demonstrate the fabrication of miniaturized OECTs with a normalized transconductance of 10.5 S cm^−1^ and an intrinsic gain of 330 V/V. These micro‐dispensed OECTs form the core of both unipolar inverter circuits, achieving a gain normalized to the supply voltage larger than 193 V^−1^ and analogue voltage amplifiers with 33 dB gain and a gain‐bandwidth product (GBW) of up to 1 10^6^ Hz. This is, to the best of our knowledge, the highest GBW reported for OECT amplifiers, highlighting the practical viability of these devices for bioelectronic applications. We also validated the effectiveness of the proposed approach by demonstrating real‐time amplification of electrooculography (EOG) signals.

## 2 Results and Discussion

### 2.1 OECT Fabrication Process and Materials

**Figure 1:**
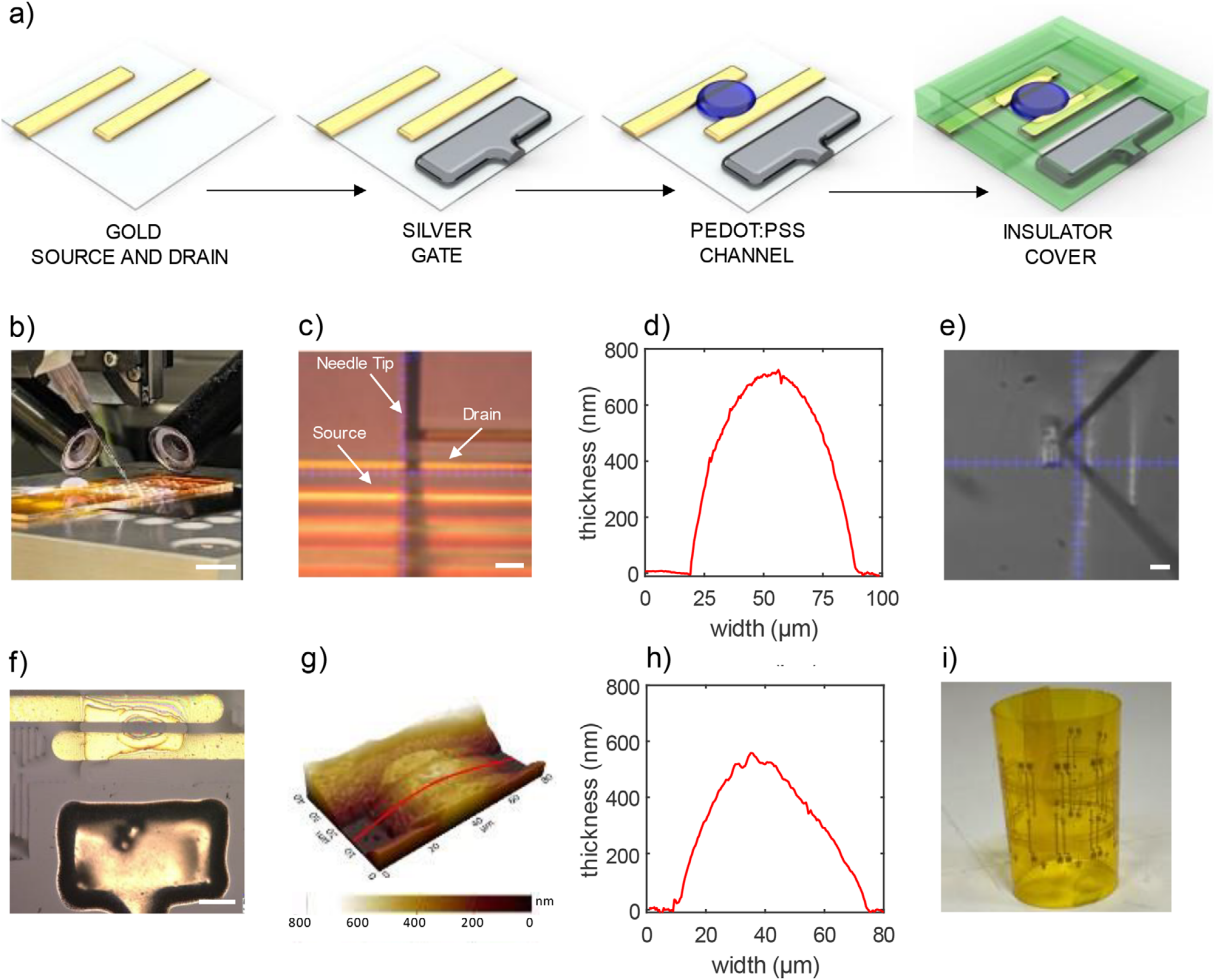
OECTs fabrication process. a) Schematic illustration of the fabrication steps and materials used in the micro‐dispensed organic electrochemical transistors (OECTs). Gold nano‐ink is employed for the source and drain electrodes, silver nanopaste for the gate electrode, PEDOT:PSS as channel material, and a UV‐curable dielectric serves as both electronic and ionic insulator over the source and drain. b) Photograph of the micro‐dispensing system, highlighting the printing needle and dual‐camera setup used for alignment and inspection. Scale bar: 1 cm. c) Micro‐dispensing of gold electrodes. Scale bar: 50 µm. d) AFM cross‐section of a printed gold electrode. e) Micro‐dispensing of a silver gate electrode. Scale bar: 100 µm. f) Optical image of a micro‐dispensed OECT. Scale bar: 50 µm. g) AFM 3D topography of a micro‐dispensed PEDOT: PSS channel connected to source and drain electrodes. h) Cross‐sectional profile of the PEDOT: PSS channel along the cutline (red line) in panel g. i) Photograph of an array of micro‐dispensed OECTs integrated on a 50 µm‐thick polyimide substrate.

The schematic structure and materials of the fabricated OECTs are schematically depicted in **Figure** **1**a. All materials were deposited and patterned using ultra‐precise micro‐dispensing onto thin (50 µm) polyimide substrates. In this technique, a functional material is loaded into a glass micro‐needle and pneumatically extruded by precisely controlling both pressure and dispensing time. We note that in the context of printed electronics and microfabrication, dispensing is often grouped within printing techniques because it allows the deposition of functional materials (e.g., conductors, insulators, adhesives) onto various substrates.^[^ ^26^, ^27^ ^]^ However, unlike conventional printing methods such as inkjet printing and aerosol printing, we show that micro‐dispensing provides advantages in terms of material viscosity, pattern resolution, and material changing. These features allowed us to combine a wide palette of materials within a single process, achieving multi‐material integration without sacrificing spatial resolution or device performance. As illustrated in Figure 1b, the micro‐needle operates in close proximity to the substrate surface, with a gantry system providing x–y–z positioning. By synchronizing needle movement with applied pressure, materials can be deposited with high spatial accuracy, enabling patterning of features down to a few micrometres in size. This versatile method supports a wide range of material viscosities, from ≈1 to 10⁶ cP. We adopted a side‐gate coplanar OECT architecture, in which gold source and drain electrodes define the channel length (L), while a silver gate electrode is positioned laterally and on the same substrate of the channel. All components are printed on the same substrate, and the electrolyte is deposited on top. Figure 1c shows a photograph when micro‐dispensing gold source and drain electrodes, while Figure 1d presents the cross‐sectional AFM profile of a printed gold line, with a width of 70 µm and a maximum height of 715 nm. For these electrodes, we used a high‐solid‐content (82 wt.%) gold nanoparticle ink with an average particle size of 112 nm and low resistivity (17.9 µΩ·cm). The channel is formed by micro‐dispensing an aqueous dispersion of poly(3,4‐ethylenedioxythiophene): polystyrene sulfonate (PEDOT:PSS), a commercially available and low‐cost organic mixed ionic‐electronic conductor, co‐formulated with dimethyl sulfoxide (DMSO) and 3‐glycidoxypropyltrimethoxysilane (GOPS). PEDOT: PSS contacts both source and drain electrodes, and its lateral dimension defines the channel width (W). Figure 1e shows the deposition of a silver gate electrode using a nano‐paste formulation with 82 wt.% solid content, particle size 35–50 nm, viscosity >100 000 cP, and resistivity 4.2 µΩ·cm. To minimize parasitic capacitance and leakage currents arising from parasitic redox interactions between the electrolyte and exposed electrodes, a dielectric material was deposited (viscosity≈25000 cP) over the source and drain areas not directly involved in the channel conduction. A second dielectric layer was added to form a microfluidic containment structure, ensuring the electrolyte remains localized over the channel and lateral gate. As an insulating layer, we used a clear solvent‐free adhesive photopolymer, particularly suitable for micro‐dispensing applications due to its precise flow properties, excellent adhesion, and chemical compatibility with the other materials employed for the OECTs. A fully printed OECT is shown in Figure 1f. AFM topography of the printed PEDOT: PSS channel connected with source and drain micro‐dispensed Au electrode is provided in Figure 1g. The PEDOT: PSS channel profile shown in Figure 1h. The channel has a width of 65 µm, an average thickness of 277 nm, and a maximum height of 553 nm. Figure 1i displays an array of fully printed OECTs monolithically integrated on a 50 µm‐thick polyimide (Kapton) substrate. To facilitate precise alignment of multilayer structures and simplify handling during fabrication, the flexible substrate was temporarily laminated onto a reusable rigid glass carrier and subsequently detached post‐fabrication. Therefore, this fabrication method is compatible with a wide range of both flexible and rigid substrates, including for example glass, silicon, and the widely used polyethylene naphthalate (PEN). Further information on the OECTs fabrication are provided in the Experimental Section, the various fabrication steps are shown in Figure S1 (Supporting Information), and the micro‐dispensing parameters for OECT fabrication are listed in Table S1 (Supporting Information).

**Figure 2:**
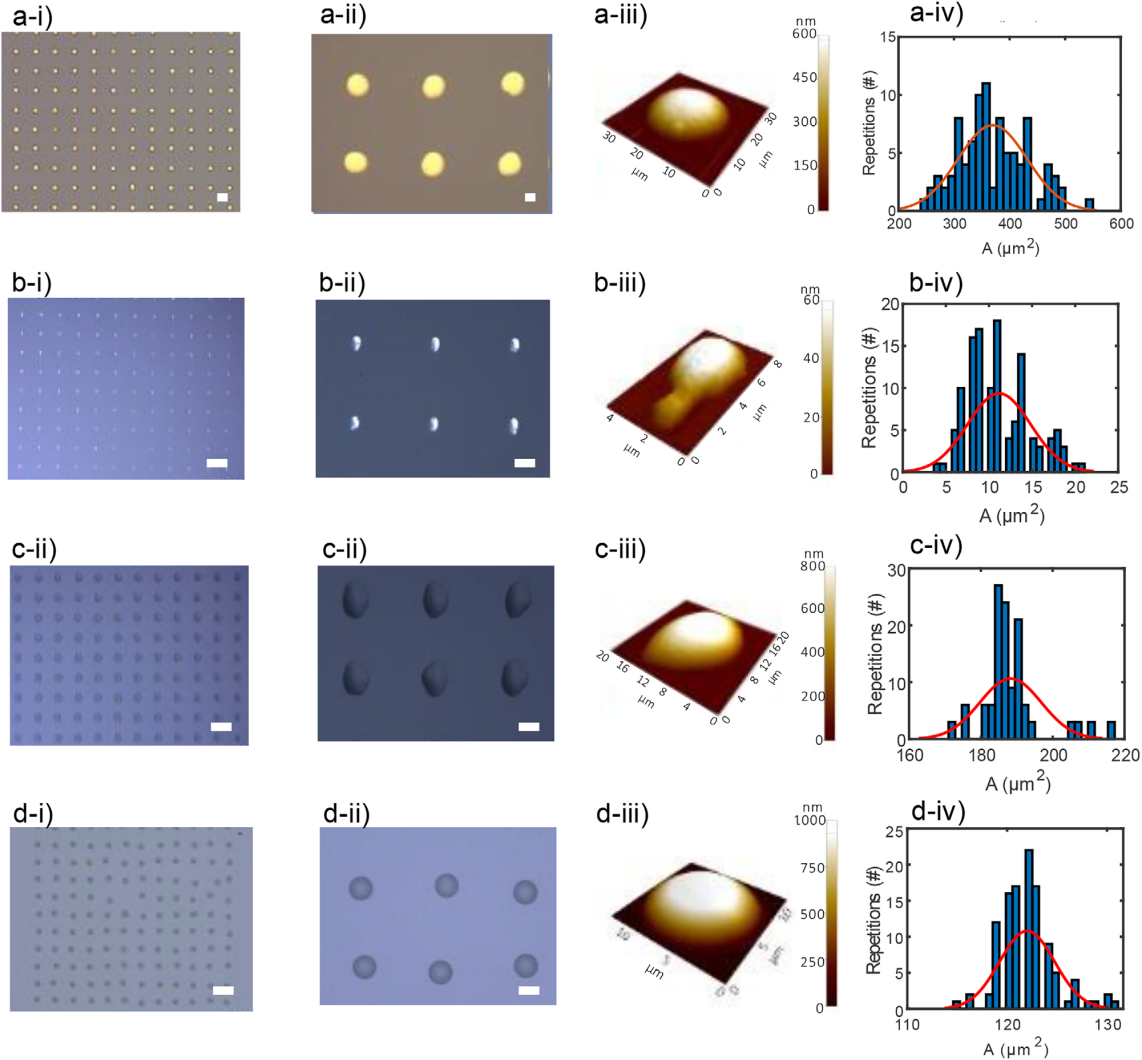
Femtoliter‐scale micro‐dispensing and analysis of micro‐dispensed material geometries. a‐i) 9×12 matrix of gold nano‐ink dots micro‐dispensed using a 5 µm inner‐diameter needle. Scale bar is 50 µm. a‐ii) Zoomed view of six Au dots. Scale bar is 10 µm. a‐iii) 3D AFM topography of a representative Au dot with a measured volume of 511 fL. a‐iv) Statistical analysis of Au dot areas measured via AFM, showing a mean area (µ_a_) of 369 µm^2^ and standard deviation (σ_a_) of 63 µm^2^. b‐i) 9 × 12 matrix of silver nano‐paste dots micro‐dispensed using a 5 µm needle. Scale bar is 50 µm. b‐ii) Zoomed view of six Ag dots. Scale bar is 10 µm. b‐iii) 3D AFM image of a single Ag dot with a printed volume of 3 fL. b‐iv) Statistical analysis of Ag dot areas, with µ_a_ = 11.2 µm^2^ and σ_a_ = 3.6 µm^2^. c‐i) 9 × 12 matrix of PEDOT: PSS dots micro‐dispensed using a 5 µm needle. Scale bar is 50 µm. c‐ii) Zoomed view of six PEDOT: PSS dots. Scale bar is 10 µm. c‐iii) 3D AFM morphology of a PEDOT: PSS dot with a volume of 85 fL. c‐iv) Statistical analysis of PEDOT: PSS dot areas, yielding µ_a_ = 188.0 µm^2^ and σ_a_ = 8.5 µm^2^. d‐i) 10 × 12 matrix of insulating material dots micro‐dispensed with a 10 µm needle. Scale bar is 50 µm. d‐ii) Zoomed view of six insulator dots. Scale bar is 10 µm. d‐iii) 3D AFM characterization of an insulator dot with a volume of 62 fL. d‐iv) Statistical analysis of insulator dot areas, with µ_a_ = 122.0 µm^2^ and σ_a_ = 2.7 µm^2^.

The proposed approach supports a broad range of functional materials, including conductors, semiconductors, insulators, and electrolytes, enabling precise deposition of multiple functional layers to obtain fully micro‐dispensed OECTs. To establish the minimum printable feature size for each material, we fabricated dot matrices using micro‐dispensing, and we performed quantitative analysis of dot area and volume. This allowed us to assess the repeatability and reliability of our fabrication process. Gold nano‐ink, used for source and drain electrodes, was dispensed using a 5 µm inner‐diameter needle to create a 9×12 dot matrix (**Figure** **2**a‐i), with a zoomed view shown in Figure 2a‐ii. AFM measurements (Figure 2a‐iii) revealed a mean dot volume of 511 fL and a lateral resolution of about 25 µm. Due to its low viscosity (≈15 cPs), the ink exhibits significant flow even at minimal pressure, setting a limit on the minimum printable volume. The distribution of dot area is shown in Figure 2a‐iv, with a mean (µ_a_) of 369 µm^2^ and standard deviation (σ_a_) of 63 µm^2^. Silver nano‐paste, used for gate electrodes, is a high‐viscosity material (∼100000 cPs), enabling high‐resolution deposition. A 9×12 matrix was printed with a 5 µm needle (Figure 2b‐i), with a detailed view of six dots in Figure 2b‐ii. AFM analysis (Figure 2b‐iii) shows a mean printed volume of 3 fL. The analysis displayed in Figure 2b‐iv yields a mean dot area of 11.2 µm^2^ with a standard deviation of 3.6 µm^2^. PEDOT: PSS, used for OECT channels, was deposited as a 9 × 12 matrix (Figure 2c‐i), with a zoom‐in displayed in Figure 2c‐ii and AFM topography of a single dot shown in Figure 2c‐iii. The mean printed volume amounts to 85 fL. The analysis shown in Figure 2c‐iv indicates a mean dot area of 188 µm^2^ and a standard deviation of 8.5 µm^2^. The insulating material, with a viscosity of ≈25 000 cPs, was micro‐dispensed using a 10 µm needle. A 10×12 dot matrix is shown in Figure 2d‐i, with a close‐up in Figure 2d‐ii. AFM analysis in Figure 2d‐iii shows a mean dot volume of 62 fL. The area distribution (Figure 2d‐iv) reports a mean of 122 µm^2^ and a standard deviation of 2.7 µm^2^. The printing parameters used for the various materials are listed in Table S2 (Supporting Information).

### 2.2 Fully Micro‐Dispensed OECTs Performance

**Figure 3:**
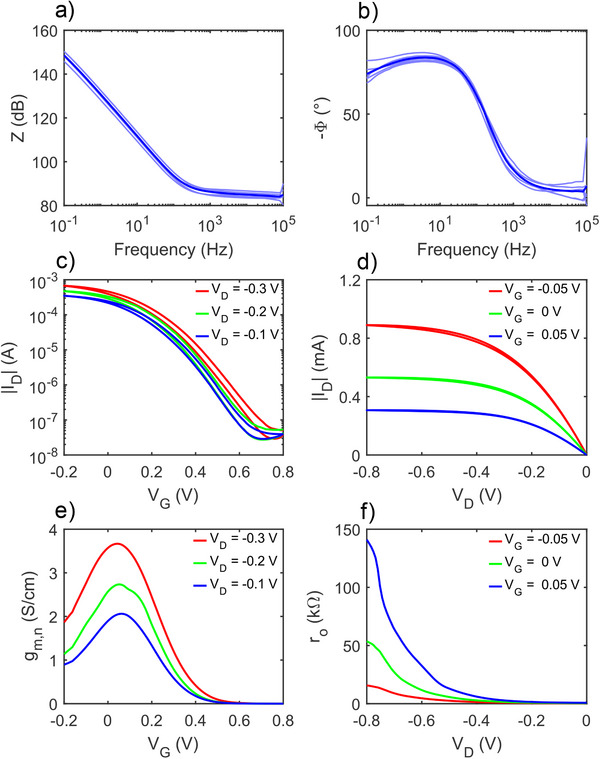
Electrical characteristics of OECTs micro‐dispensed on polyimide substrates. a) Impedance magnitude and b) phase obtained from EIS measurements of six nominally identical OECTs (thin lines). The thick blue line represents the average response. EIS measurements were carried out using a DC bias V_DC_ = 0 V and an AC voltage amplitude V_AC_ = 10 mV, in 0.1 m NaCl aqueous electrolyte. c) Average transfer characteristics of six OECTs measured at various drain voltages V_D_ = −0.1, −0.2, and −0.3 V. d) Average output characteristics of six OECTs measured at various gate voltages V_G_ = −0.05, 0, and 0.05 V. [R3Q2] e) Transconductance g_m_ = dI_D_/dV_G_ normalized to the channel dimensions g_m,n_ = g_m_/(W L t) as a function of V_G_ for various drain voltages. f) Output resistance r_o_ as a function of V_D_ for various gate voltages. The output resistance r_o_ = (dI_D_/dV_D_)^−1^ is obtained from the measured output characteristics. The measurements were performed in 1 M NaCl aqueous electrolyte. OECTs were fabricated on a 50 µm thick polyimide substrate. The channel geometries of the OECTs are: width W = 60 µm, length L = 12 µm, and average thickness t ≈ 750 nm.

Since OECTs are volumetric iontronic devices, the deposited volume of PEDOT: PSS is a critical parameter for ensuring uniformity and reproducibility. To evaluate the variability of the micro‐dispensed channel, we performed Electrochemical Impedance Spectroscopy (EIS) measurements on several nominally identical OECTs. EIS measurements were performed in a two‐electrode configuration, using the gate electrode as both reference and counter electrode. **Figure** **3**a,b shows the measured impedance magnitude and phase (thin light‐blue lines) along with their averages (thick blue lines) as a function of frequency. We modeled the EIS spectra using the Randles equivalent circuit, composed of a resistor in series with a capacitor. In this model, the resistor represents the ionic resistance of the electrolyte, while the capacitor describes the ionic‐electronic charge interaction within the polymer volume.^[^ ^34^ ^]^ The volume V of the channel was independently measured using AFM. Then, we calculated the volumetric capacitance C_V_ = C/V, where C is the capacitance obtained from the EIS spectra. The extracted capacitance C and the corresponding volumetric capacitance C_V_ are displayed in Figure S2 (Supporting Information). We obtained an average volumetric capacitance of 36.45 ± 0.87 F·cm^−3^ and a micro‐dispensed polymer volume of 1.25 10^−9^ ± 0.03 10^−9^ cm^3^. The consistency of these results confirms the uniform deposition of the PEDOT: PSS channel using the micro‐dispensing technique.

The average transfer characteristics (I_D_–V_G_) of various OECTs are presented in Figure 3c for various drain voltages (V_D_ = −0.1, −0.2, and −0.3 V). Figure S3 (Supporting Information) shows the measured transfer characteristics of six OECTs (thin lines). When a positive gate voltage (V_G_) is applied, cations drift into the polymer, reduce the hole concentration and lower the drain current (I_D_). Conversely, when a negative V_G_ is applied, the previously injected cations leave the polymer, and anions enter the channel, increasing the hole concentration and the drain current. The OECTs display an on‐current of about 1 mA and an off‐current of about 20 nA, resulting in an on/off ratio of approximately 10⁵. The I_D_‐V_G_ measurements of the various OECTs are well aligned and demonstrate reproducible electrical characteristics across all tested devices. To assess the performance when using a silver gate, we conducted a comparative study of OECTs gated with a micro‐dispensed Ag electrode and with a conventional Ag/AgCl pellet. As shown in Figure S4 (Supporting Information), the I_D_–V_G_ curves are almost perfectly overlapped, and the impedance spectra are in excellent agreement across the frequency range. The slight increase in impedance observed at frequencies above 200 Hz can be attributed to the significant size difference between the two gate electrodes. The micro‐dispensed Ag gate has micrometer‐scale dimensions, while the Ag/AgCl pellet is millimeter‐sized. This disparity in surface area leads to a modest increase in ionic resistance, which is expected. These results confirm that the Ag gate used in our devices provides functional equivalence to Ag/AgCl in terms of gating performance.

Long‐term stability measurements are displayed in Figure S5 (Supporting Information). The micro‐dispensed OECTs maintain an on/off current ratio greater than 10^3^, with the on current showing a decrease of approximately 19%, and the off current decreasing by about 13% over the course of the test. Importantly, much of this drift occurs within the first 20 minutes, after which the current levels stabilize, indicating stable and reliable performance under continuous operation. These results confirm the robustness of the fabricated OECTs under repeated electrical stress, further supporting their potential for long‐term applications in bioelectronics and sensing technologies. Output characteristics (I_D_–V_D_) of micro‐dispensed OECTs are shown in Figure 3d for various gate voltages (V_G_ = −0.05, 0, and 0.05 V). The curves reveal the expected transition from the linear to the saturation regime, with the transition point clearly dependent on the applied V_G_. Figure 3e shows the transconductance g_m_ = dI_D_/dV_G_ normalized to the channel dimensions g_m,n_ = g_m_/(W L t), for various drain voltages. From the average I_D_–V_G_ curve measured at V_D_ = −0.3 V, the peak normalized transconductance reaches approximately 3.7 S cm^−1^ at V_G_ = 0.1 V, which is in line with state‐of‐the‐art OECT performance. The output resistance r_o_ = (dI_D_/dV_D_)^−1^ obtained from the measured output characteristics is shown in Figure 3f. r_o_ increases with decreasing drain voltage and increasing gate voltage, reaching a peak value of approximately 140 kΩ at the operating point V_D_ = −0.8 V and V_G_ = 0.05 V. A parametric analysis of I_D_‐V_G_, I_D_‐V_D,_ and the corresponding g_m_ and r_o_ as a function of applied V_G_ and V_D_ is displayed in Figure S6 (Supporting Information).

OECTs are inherently volumetric iontronic devices, where ionic‐electronic interactions within the semiconducting channel govern their performance. This operation principle makes the performance of OECTs highly sensitive to the properties of the electrolyte, including its ionic concentration. Understanding and tuning this electrolyte‐dependent response is critical for achieving optimal device performance, particularly in applications where high amplification and low‐voltage operation are required, such as biopotential recording and biosensing systems. The figure of merit that determines the intrinsic amplification of a transistor is the intrinsic gain that depends on both transconductance and output resistance, and reads: G_I_ = g_m_ × r_o_.^[^ ^35^, ^36^ ^]^ Consequently, as a first step, to explore and enhance the transistor amplification, we investigated the impact of the electrolyte ion concentration on the OECT electrical characteristics.

**Figure 4:**
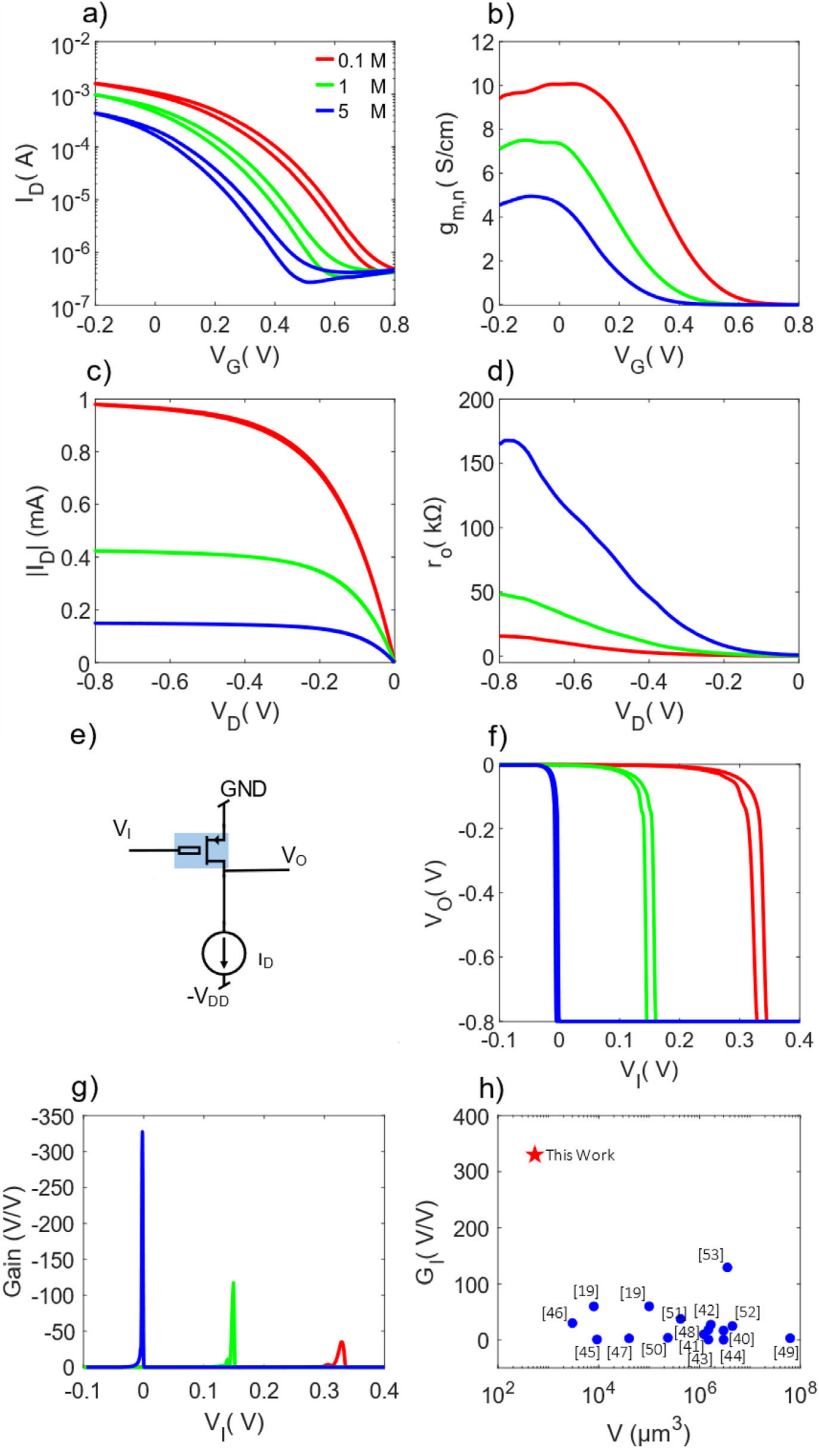
Electrical characterization versus ion concentration of micro‐dispensed OECTs printed on polyimide substrate. a) Transfer characteristics measured with various ion concentrations and b) corresponding normalized transconductance g_m,n_. Applied drain voltage is V_D_ = ‐0.8 V, which corresponds to the regime of maximum transconductance. c) Output characteristics measured at V_G_ = 0 V, which corresponds to the condition of maximum gain, with various ion concentration and d) corresponding output resistance r_o_. e) Schematic circuit of the OECT current‐driven configuration. f) Measured V_O_‐V_I_ characteristic at a current bias I_D_ = 200 µA and various ion concentration, and g) corresponding gain dV_O_/dV_I_. h) Comparison of the intrinsic gain of the OECTs fabricated with additive manufacturing techniques versus the channel volume (see also Table S3 and Figure S7, Supporting Information).

The transfer characteristics measured at various ion concentrations (0.1, 1, and 5 M NaCl solution) are presented in **Figure** **4**a. Increasing the ion concentration, the transfer curve shifts closer to zero gate (V_G_ ≈ 0 V), which is advantageous for low‐power applications. The corresponding normalized transconductance are displayed in Figure 4b. More in detail, the g_m_ peak shifts with the ion concentration, and the maximum transconductance reduces with increasing ion concentration, according to previous studies.^[^ ^37^ ^]^ The output characteristics and the corresponding output resistance are displayed in Figure 4c,d, respectively. We found that r_o_ increases with both the electrolyte ion concentration and the applied drain voltage. To experimentally obtain the transistor gain, we operated the OECT in a current‐driven configuration, schematically depicted in Figure 4e. In this architecture, the OECT is connected in series with a current source (I_B_), the input voltage (V_I_) is applied to the gate terminal (V_G_ = V_I_), and the output voltage (V_O_) is measured at the drain terminal (V_D_ = V_O_). Figure 4f shows the V_O_–V_I_ transfer characteristics of a current‐driven OECT operated at I_D_ = 200 µA and V_DD_ = 0.8 V, under various NaCl concentrations. At negative V_I_, the OECT operates in the linear regime: anions are injected into the PEDOT: PSS channel, increasing hole conductivity, and V_O_ ≈ 0 V. As V_I_ increases, cations are progressively injected, de‐doping the polymer and increasing r_o_, because the OECT operates in saturation. This leads to a sharp transition of V_O_ from GND to –V_DD_ and, in this region, the variation of V_O_ with respect to V_I_, (dV_O_/dV_I_) corresponds to the OECT intrinsic gain G_I_ = g_m_ × r_o_.^[^ ^38^ ^]^ Figure 4g shows the measured gain as a function of the ion concentration. As the ion concentration is increased from 0.1 to 5 M, the gain increases from −34 V/V to −330 V/V. Concurrently, the transition voltage, defined as the input voltage V_I_ at which V_O_ = –V_DD_, shifts closer to 0 V, further supporting low‐voltage circuit operation.

In light of the previous analysis, this can be explained as follows. Increasing the ion concentration, the OECT threshold voltage shifts to less positive voltages (Figure 4a), the OECT saturation takes place at smaller V_I_, and this is mirrored in a shift of the V_O_‐V_I_ characteristic transition point (Figure 4f). g_m_ is maximum when the transition voltage (V_I_ = V_G_) is closed to zero and reduces at smaller or larger voltages (Figure 4b), while r_o_ increases when V_D_ reduces (Figure 4d) providing an enhanced r_o_ when V_O_ approaches ‐V_DD_ (V_O_ = V_D_). Notably, in the circuit shown in Figure 4e, the supply voltage V_DD_ was set to −0.8 V. In this configuration, the maximum gain is achieved at the input voltage at an input voltage V_I_ = 0 V, as indicated by the blue line in Figure 4f. It is worth noting that in this current‐driven amplifier configuration, both the current source and the supply voltage V_DD_ can be independently biased. For example, in our previous work^[^ ^37^, ^39^ ^]^ we leveraged this flexibility to overcome the fundamental trade‐off between sensitivity, operational range, and power supply limitations. In Figure 4h the gain as a function of the polymer channel volume is benchmarked against various OECT materials and technologies. A detailed comparative overview of fully printed OECT performance – including materials, structures, geometries, and key transistor parameters – is presented in Table S3 (Supporting Information). The comparison includes OECTs fabricated with several additive manufacturing techniques (e.g., inkjet printing^[^ ^40^, ^41^ ^]^ and screen printing^[^ ^42^, ^43^ ^]^) and both n‐type and p‐type OMIEC materials, such as BBL^[^ ^19^ ^]^ PEDOT: PSS,^[^ ^44^, ^45^ ^]^ and pgTTT.^[^ ^46^ ^]^ The comparison shows that our micro‐dispensed OECTs exhibit downscaled geometries while achieving the highest intrinsic gain reported so far for fully printed devices (Figure 4h; Figure S7 in Supporting Information). This performance is attributed to the synergistic effects of precise material geometry control during fabrication and strategic tuning of the OECT parameters. The unique combination of high gain, scalable micro‐dispensing, and tunable electrical behavior makes these OECTs ideal devices for biosignal amplification.

### 2.3 Biosignal Amplification with Monolithically Integrated OECT Amplifiers

**Figure 5:**
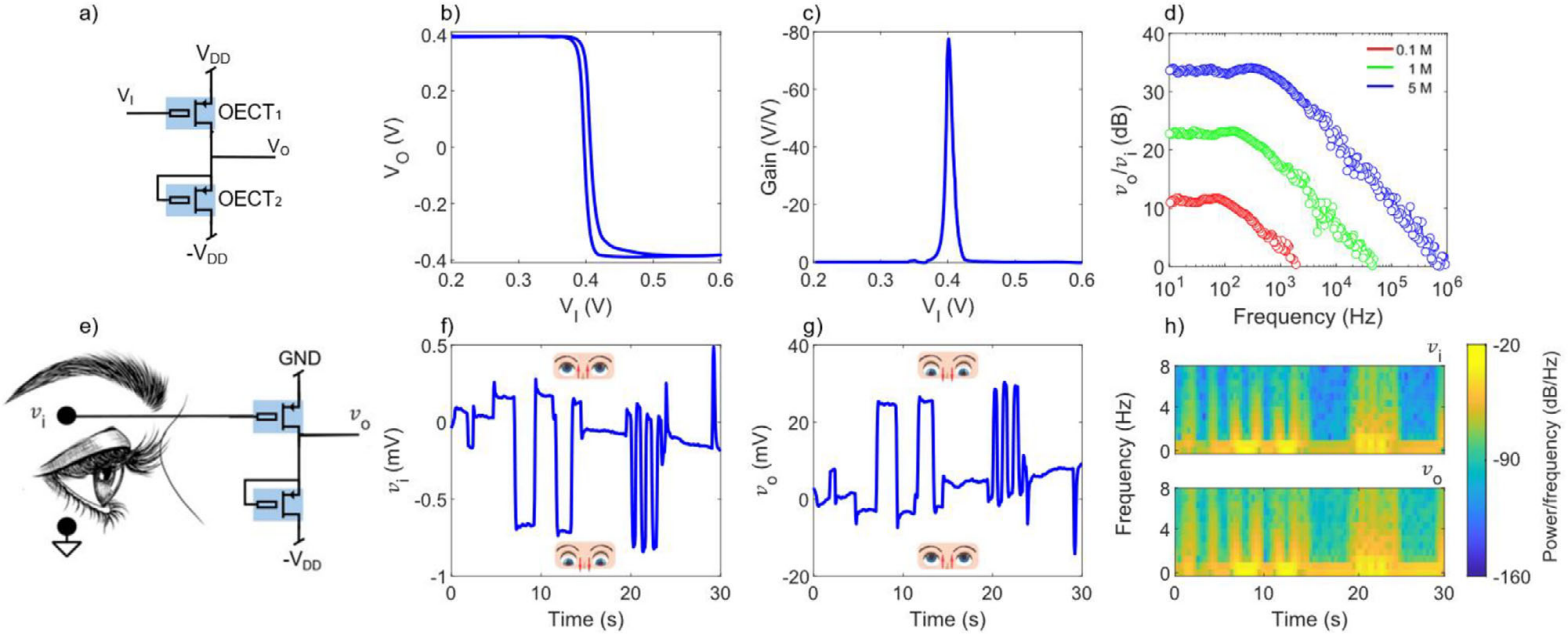
Micro‐dispensed OECT amplifier for electro‐oculography. a) Schematic circuit diagram of a unipolar 0‐V_GS_ amplifier. b) Measured voltage transfer characteristic V_O_‐V_I_ of an OECT amplifier and c) corresponding voltage gain. d) Bode diagram of the unipolar OECT amplifier, using 5, 1, and 0.1 M ion concentration. e) Schematic circuit of the EOG measurement set‐up. f) Measured EOG signal of vertical eye movement as input of the unipolar OECT amplifier, with 5 M ion concentration. g) Real‐time amplified output signal. h) Spectrograms of the input and output signals of the OECT amplifier. The OECT geometries are the following: W_1_ = 65 µm, L_1_ = = 12 µm, W_2_ = 200 µm, and L_2_ = 12 µm.

High‐gain transistors are essential for developing high‐performance amplifiers capable of enhancing weak biological signals – such as those in electrophysiology – without requiring complex circuitry or high‐voltage supplies. In this context, we exploit our micro‐dispensed OECTs to implement a unipolar voltage amplifier suitable for low‐voltage bioelectronic signal amplification. As displayed in **Figure** **5**a, the amplifier configuration is built using two OECTs arranged in series: a driver and a load to obtain an active‐load common‐source amplifier. This architecture resembles that of an OECT‐based inverter, which is a fundamental circuit topology allowing integration of both digital and analogue circuits.^[^ ^15^, ^38^ ^]^ We operated this circuit configuration in the analogue domain, acting as a linear voltage amplifier rather than a digital logic gate. More in detail, the input voltage V_I_ is applied to the gate of the driver OECT_1_, while the load OECT_2_ operates in a zero‐V_GS_ configuration as its gate is shorted to the source, ensuring low power consumption. The supply voltage V_DD_ is applied to the source of the driver OECT_1_, and the output voltage V_O_ is measured at the node connecting the drain of the driver OECT_1_ and the source of the load OECT_2_. This topology was selected specifically to minimize external instrumentation, which is advantageous for compact and low‐cost applications, such as EOG signal acquisition. This circuit, composed of two OECTs, does not require a separate current source, but only a supply voltage, thereby simplifying the system.

The voltage transfer characteristic (V_O_–V_I_) of the fabricated amplifier is shown in Figure 5b. When V_I_ is low (e.g., equal to −V_DD_), the gate‐source voltage (V_SG_) of the OECT_1_ is large, and the transistor operates in a high‐conductance state, pulling V_O_ close to V_DD_. As V_I_ increases, the conductivity of the driver OECT_1_ decreases, leading to a progressive voltage drop across the load. Near the transition region, a steep change in V_O_ occurs, and the gain of the amplifier, defined as dV_O_/dV_I_, reaches its peak. As V_I_ continues to increase, the driver eventually turns off, and V_O_ approaches −V_DD_. The gain curve displayed in Figure 5c highlights a maximum voltage gain of 77.5, which corresponds to a normalized gain G_n_ = Gain/V_DD_ of 193.7 V^−1^ – the highest value reported to date for fully printed OECT‐based amplifiers. **Table** **1** benchmarks our OECT amplifier monolithically integrated with micro‐dispensing against the state‐of‐the‐art of unipolar and complementary amplifiers based on inverter topologies, focusing on topology, fabrication method, supply voltage (V_DD_), and gain normalized to the supply voltage (G_n_). The comparison shows that the majority of previously reported OECT‐based approaches adopt a unipolar resistive load topology, commonly implemented via screen printing or hybrid approaches that combine screen printing with inkjet printing, aerosol jet, or spray coating. These OECT amplifiers operate at relatively high supply voltages (ranging from to 5 V) and display modest normalized gain values, often below 20 V^−1^, despite utilizing advanced and multi‐step fabrication processes. Complementary topologies, which integrate both p‐type and n‐type OECTs, offer good performance in some cases. For instance, the OECT amplifier in ref.[19] achieves a normalized gain of 47.14 V^−1^. However, such designs typically involve increased fabrication complexity, material requirements, and challenges in device balancing.^[^ ^38^ ^]^ In contrast, our approach entirely based on micro‐dispensing, allows precise material deposition with minimal complexity, providing high gain and low‐voltage OECT amplifiers.

**Table 1:** Performance of OECT amplifiers based on inverter‐like configurations and fabricated with additive manufacturing methods.

Topology	Fabrication method	Gain [V/V]	V_DD_ [V]	G_n_ [V^−1^]	Refs.
Unipolar resistive load	Screen printing	30	1.5	20	[54]
Unipolar 0‐V_GS_ load	Capillary printing	82	0.6	136.67	[47]
Unipolar resistive load	Screen printing and aerosoljet printing	5.8	1	5.8	[28]
Complementary	Screen printing and spray coating	33	0.7	47.14	[19]
Unipolar resistive load	Screen printing and inkjet printing	5.8	1	5.8	[46]
Unipolar resistive load	Screen printing	13.93	4	3.48	[55]
Unipolar 0‐V_GS_ load	Dispensing	0.25	0.3	0.83	[53]
Unipolar (3T design) load	Screen printing	43	2.5	17.2	[56]
Unipolar resistive load	Screen printing	5	5	1	[54]
Unipolar resistive load	Screen printing	3.5	3.5	1	[57]
Unipolar 0‐V_GS_ load	Micro‐dispensing	77.5	0.4	193.7	This Work

To deeper our study, we investigated the frequency response of the micro‐dispensed OECT amplifiers. Representative Bode plots measured at various ion concentration are presented in Figure 5d. As the ion concentration increased, the amplifier performance improved significantly. At the lowest tested concentration of 0.1 M, the voltage gain was approximately 11 dB. This increased to about 24 dB at 1 M, and reached 33 dB at 5 M, which corresponds to about ×45 amplification (Figure S8, Supporting Information). At this concentration, we achieved a gain‐bandwidth product of 1 MHz and a maximum signal‐to‐noise ratio (SNR) greater than 30 dB. Moreover, as shown in Figure S9 (Supporting Information), the amplifier presents a SNR that remains consistently above 10 dB across the 1 Hz to 1 MHz frequency range. These figures of merit confirm the effectiveness of high‐gain micro‐dispensed OECT amplifiers. [R3Q13] It is worth noting that the GBW is rarely reported in the literature, despite its importance in evaluating both transconductance and operating speed. A notable exception is the work by Cea et al.,^[^ ^20^ ^]^ in which enhancement‐mode, internally ion‐gated OECTs fabricated via photolithography achieved a GBW of 638 kHz. This value is approximately half of what we obtained using our precise micro‐dispensing fabrication method, underscoring the effectiveness of our approach in realizing high‐performance devices.

To demonstrate the practical relevance of our approach, we applied the micro‐dispensed unipolar OECT amplifier to amplify electrooculogram (EOG) signals. EOG signals originate from the corneoretinal potential (CRP), a natural electric dipole between the positively charged cornea and the negatively charged retina. These signals typically have amplitudes ranging from 0.2 to 1.0 mV and a frequency content between 0 and 30 Hz, making them among the weakest non‐invasively measurable bioelectrical signals. High‐gain amplification is therefore essential for their reliable detection. At the state of art, only a limited number of studies have explored the application of OECTs for electrooculography measurements.^[^ ^58^, ^59^ ^]^ These efforts have primarily relied on parylene‐C‐based photolithographic fabrication methods, with the exclusion of integrated gate electrodes, and are restricted to rigid substrates. Such constraints significantly hinder their suitability for seamless integration into embedded or wearable systems. More in detail, Leleux et al. were the first to implement EOG detection based on OECT.^[^ ^58^ ^]^ Their work showed that a transconductance of 1.3 mS enabled a nearly tenfold amplification when converting the drain current across an external resistor. More recently, Wu et al. advanced this approach by developing complementary inverter OECTs based on an ambipolar ladder‐type polymer heterojunction composed of 6H‐pyrrolo[3,2‐b:4,5‐b’]bis[1,4]benzothiazine (PBBTL) mixed with poly(benzimidazobenzophenanthrolinedione) (BBL), reaching a maximum gain of 42 V/V.^[^ ^59^ ^]^

In our work, focusing on the miniaturization and fully‐additive mask‐less OECT integration, we demonstrate OECT‐based amplifiers with a voltage gain of 77.5 V/V at a supply voltage V_DD_ = 0.4 V, allowing high fidelity real‐time EOG measurements. In the experimental setup shown in Figure 5e, vertical eye movement signals were acquired using skin patch electrodes. To maintain a low‐power operation, no external voltage bias was applied to the input of the amplifier: the gate of the driver OECT was directly connected to the electrode, and the source was grounded. This configuration capitalizes on the OECT amplifier high gain even under zero gate bias, maximizing power efficiency and simplicity. The raw recorded signals of vertical eye movements are shown in Figure 5f, where upward and downward gaze shifts are clearly visible as positive and negative voltage deflections, respectively. The maximum biosignal amplitude is less than 0.7 mV. Figure 5g displays the corresponding amplified output, showing signal peaks up to 32 mV. The experimental setup and real‐time recordings are further illustrated in Movie S1 (Supporting Information). Finally, we evaluated the frequency content of the input and output signals over time using spectrograms, as shown in Figure 5h. The dominant frequencies remained stable throughout the measurement period, while the output signal exhibited significantly higher power. Additional time‐domain recordings of input and output biosignals are shown in Figure S10 (Supporting Information), underscoring the consistent performance of the amplifier even at reduced ion concentrations.

## 3 Conclusion

In this work, we demonstrate the monolithic integration of high‐performance OECTs and amplifier circuits fabricated entirely through micro‐dispensing on flexible substrates. Our approach enables precise additive patterning of diverse functional materials, including conductors, semiconductors, electrolytes, and insulators, with femtoliter control and micrometer resolution. This required careful engineering of the dispensing parameters to achieve single‐process multilayer stacking with micrometer‐scale resolution of materials with viscosities ranging from 10 to 100 000 cP. This versatility is crucial for achieving multi‐material integration without sacrificing spatial resolution or device performance. The printed transistors exhibit an intrinsic gain of 330 V/V, among the highest reported for printed OECTs, indicating exceptional signal amplification capability. Achieving this level of performance in OECTs fabricated with a fully additive manufacturing method represents a significant step forward in both device fabrication and material integration. Moreover, the proposed approach allowed the demonstration of monolithically integrated amplifier circuits. These circuits achieve a maximum voltage gain of 77.5 and a supply‐normalized gain of 193.7 V^−1^, with a GBW of 1 MHz, enabling broadband bio‐signal amplification. Specifically, real‐time acquisition and amplification of EOG signals with minimal distortion is demonstrated with OECT circuit‐level integration.

The proposed micro‐dispensing approach can be applied to a wide range of materials, including OMIECs,^[^ ^3^ ^]^ printable solid electrolytes,^[^ ^50^, ^60^ ^]^ and emerging bioresorbable or biodegradable inks,^[^ ^8^, ^61^ ^]^ as well as OECT architectures, including for example, vertical OECTs,^[^ ^9^, ^18^ ^]^ and floating‐gate OECTs.^[^ ^31^ ^]^ Prospectively, our work opens pathways toward microscale integrated OECT‐based bioelectronics,^[^ ^25^, ^62^ ^]^ biosensing,^[^ ^63^, ^64^ ^]^ and neuromorphic interfacing.^[^ ^65^, ^66^, ^67^ ^]^ Moreover, the proposed approach could be extended to larger arrays and more complex multi‐transistor circuits, opening opportunities for scalable bioelectronic integration.

## 4 Experimental Section

### Materials

Ag Nanopaste and Gold Nanoink were acquired from XTPL. Insulating material NOA68TH was acquired from Norland. Needles with inner diameter dimensions ranging from 20 to 5 µm were supplied by XTPL. Glass substrates microscope slides were supplied by Epredia. Flexible polyimide (Kapton) substrates with a thickness of 50 µm were acquired from Sigma‐Aldrich. PEDOT: PSS (PH500) aqueous solution was acquired from Clevios^TM^, Heraeus. PEDOT: PSS was mixed with DMSO 5% vol, and 1 vol% (3‐glycidyloxypropyl) trimethoxysilane (GOPS). The solution was vigorously stirred overnight and filtered with a 0.22 µm cellulose filter. The prepared polymer blend was kept at 4 °C before use. Dimethyl sulfoxide (DMSO) and 3‐glycidoxypropyltrimethoxysilane (GOPS) were acquired from Sigma‐Aldrich and used without further purification.

### Printing System

All the printing steps are performed using XTPL Delta printing system, that requires commercial software packages (AutoCAD, Notepad++) to follow CAD drawings and scripts generated in XTPL language. The printer system is based on a three‐axial micrometric (nanometric in z axis) control of motion and on real‐time fine control of the pressure applied to the nozzle to control the ink flow. The high‐resolution printing, including channel, gate, electrodes and covering layer, was performed using a glass needle with an inner diameter (ID) ranging from 5 to 20 µm.

### Printing Parameters

For all the material micro‐dispensed with XTPL Delta printing system there are four printing parameters. Specifically, the dispensing velocity (V_X_, V_Y_ and V_Z_) along the three dimensions, the pressure (P_ON_ and P_OFF_) that controls the flow of the ink with an on pressure and an off pressure, the standard delay (SD) is the waiting time between the P_ON_ action and the start of the print movement, and the Z‐heigh movement (Z_H_) is the distance between the needle and the substrate (along the z‐axis) when the ink is not dispensed. Here, the parameters of each material dispensed are provided. Ag Nanopaste: Needle ID = 5 µm, V_X_ = V_Y_ = 0.4 mm/s, V_Z_ = 6 mm/s, P_ON_ = 6500 mbar, P_OFF_ = 1500 mbar, SD = 1500 ms, Z_H_ = 100 µm. Gold nanoink: Needle ID = 15 µm, V_X_ = V_Y_ = 1 mm/s, V_Z_ = 6 mm/s, P_ON_ = 1 mbar, P_OFF_ = 1 mbar, SD = 0 ms, Z_H_ = 50 µm. Insulator: needle ID = 10 µm, V_X_ = V_Y_ = 0.2 mm/s, V_Z_ = 6 mm/s, P_ON_ = 1500 mbar, P_OFF_ = 250 mbar, SD = 100 ms, Z_H_ = 50 µm. Polymer‐blend: needle ID = 0.5 µm, V_X_ = V_Y_ = 1 mm/s, V_Z_ = 6 mm/s, P_ON_ = 10 mbar, P_OFF_ = 10 mbar, SD = 10 ms, Z_H_ = 30 µm.

### OECTs and OECT Amplifier Fabrication

As a first step, the substrate was cleaned by sonication with acetone, isopropyl alcohol, HPLC water for 5 min each and treated with UV ozone cleaner for 5 min. Then the gold electrodes were printed and baked 30 min at 250 °C. After, silver gate and connections were deposited and baked 10 min at 200 °C. OMIEC channel was printed and baked 30 min at 140 °C. After, insulator layer was printed and cured in three different times, the first is the fast curing necessary to fix the geometry of the printed layer, after a slow curing for 12 h at room temperature, and finally the layer was baked at 100 °C for 10 min. This fabrication process avoids the diffusion of the fine insulator, thus increasing the printing resolution.

### Electrical Characterization

The DC electrical characteristics were measured with source measure unit (SMU) Keythley 2636A. EIS were performed with PalmSense 4, in a two‐electrodes configuration, where the gate electrode was used as counter/reference electrode, while source and drain electrodes were shorted together, and the OECT channel was used as working electrode.

### AFM Profilometry

AFM profilometries were made using NX10 Park System in non‐contact mode with a NSG30 cantilever produced by TipsNano with tip curvature radius of 10 nm.

### Biosignals Measurements

All participants provided written informed consent prior to their involvement in the study, which was conducted in accordance with institutional ethical guidelines.

## Conflict of Interest

The authors declare no conflict of interest.

## Supporting information



Supporting Information

Supplemental Movie 1

## Data Availability

The data that support the findings of this study are available from the corresponding author upon reasonable request.

## References

[1] J. Rivnay , S. Inal , A. Salleo , R. M. Owens , M. Berggren , G. G. Malliaras , Nat. Rev. Mater. 2018, 3, 1.

[2] H. Kim , Y. Won , H. W. Song , Y. Kwon , M. Jun , J. H. Oh , Adv. Sci. 2024, 11, 2306191. 10.1002/advs.202306191 PMC11251567 38148583

[3] B. D. Paulsen , K. Tybrandt , E. Stavrinidou , J. Rivnay , Nat. Mater. 2020, 19, 13. 31427743 10.1038/s41563-019-0435-z

[4] A. Savva , R. Hallani , C. Cendra , J. Surgailis , T. C. Hidalgo , S. Wustoni , R. Sheelamanthula , X. Chen , M. Kirkus , A. Giovannitti , A. Salleo , I. McCulloch , S. Inal , Adv. Funct. Mater. 2020, 30, 1907657.

[5] Y. Yao , W. Huang , J. Chen , X. Liu , L. Bai , W. Chen , Y. Cheng , J. Ping , T. J. Marks , A. Facchetti , Adv. Mater. 2023, 35, 496. 10.1002/adma.202209906 36808773

[6] L. M. M. Ferro , L. Merces , D. H. S. de Camargo , C. C. Bof Bufon , Adv. Mater. 2021, 33, 2170223. 10.1002/adma.202101518 34061409

[7] M. Abarkan , A. Pirog , D. Mafilaza , G. Pathak , G. N'Kaoua , E. Puginier , R. O'Connor , M. Raoux , M. J. Donahue , S. Renaud , J. Lang , Adv. Sci. 2022, 9, 1831. 10.1002/advs.202105211 PMC8922095 35064774

[8] M. Wu , K. Yao , N. Huang , H. Li , J. Zhou , R. Shi , J. Li , X. Huang , J. Li , H. Jia , Z. Gao , T. H. Wong , D. Li , S. Hou , Y. Liu , S. Zhang , E. Song , J. Yu , X. Yu , Adv. Sci. 2023, 10, 2300504. 10.1002/advs.202300504 PMC10190644 36825679

[9] D. A. Koutsouras , F. Torricelli , P. W. M. Blom , Adv. Electron. Mater. 2023, 9, 2200868.

[10] J. E. Tyrrell , K. Petkos , E. M. Drakakis , M. G. Boutelle , A. J. Campbell , Adv. Funct. Mater. 2021, 31, 2103385.

[11] J. Rivnay , P. Leleux , M. Ferro , M. Sessolo , A. Williamson , D. A. Koutsouras , D. Khodagholy , M. Ramuz , X. Strakosas , R. M. Owens , C. Benar , J. M. Badier , C. Bernard , G. G. Malliaras , Sci. Adv. 2015, 1, 1400251. 10.1126/sciadv.1400251 PMC4640642 26601178

[12] I. Krauhausen , C. Coen , S. Spolaor , P. Gkoupidenis , Y. van de Burgt , Adv. Funct. Mater. 2024, 34, 2307729.

[13] H. Liu , J. Song , Z. Zhao , S. Zhao , Z. Tian , F. Yan , Adv. Sci. 2024, 11, 2305347. 10.1002/advs.202305347 PMC11251571 38263718

[14] J. Wang , S. Lee , T. Yokota , T. Someya , Adv. Funct. Mater. 2022, 32, 2200458.

[15] P. Romele , M. Ghittorelli , Z. M. Kovács‐Vajna , F. Torricelli , Nat. Commun. 2019, 10, 3044. 31292452 10.1038/s41467-019-11073-4 PMC6620344

[16] F. Bonafè , F. Decataldo , I. Zironi , D. Remondini , T. Cramer , B. Fraboni , Nat. Commun. 2022, 13, 5423. 36109508 10.1038/s41467-022-33094-2 PMC9477811

[17] K. Lieberth , P. Romele , F. Torricelli , D. A. Koutsouras , M. Brückner , V. Mailänder , P. Gkoupidenis , P. W. M. Blom , Adv. Healthcare Mater. 2021, 10, 2100845. 10.1002/adhm.202100845 PMC11468701 34309226

[18] W. Huang , J. Chen , Y. Yao , D. Zheng , X. Ji , L. W. Feng , D. Moore , N. R. Glavin , M. Xie , Y. Chen , R. M. Pankow , A. Surendran , Z. Wang , Y. Xia , L. Bai , J. Rivnay , J. Ping , X. Guo , Y. Cheng , T. J. Marks , A. Facchetti , Nature 2023, 613, 496. 36653571 10.1038/s41586-022-05592-2 PMC9849123

[19] C. Y. Yang , D. Tu , T. P. Ruoko , J. Y. Gerasimov , H. Y. Wu , P. C. Harikesh , M. Massetti , M. A. Stoeckel , R. Kroon , C. Müller , M. Berggren , S. Fabiano , Adv. Electron. Mater. 2022, 8, 2100907.

[20] C. Cea , G. D. Spyropoulos , P. Jastrzebska‐Perfect , J. J. Ferrero , J. N. Gelinas , D. Khodagholy , Nat. Mater. 2020, 19,679. 32203456 10.1038/s41563-020-0638-3

[21] S. Han , S. Yamamoto , A. G. Polyravas , G. G. Malliaras , Adv. Mater. 2020, 32, 2004790. 10.1002/adma.202004790 33118196

[22] S. Zhang , E. Hubis , G. Tomasello , G. Soliveri , P. Kumar , F. Cicoira , Chem. Mater. 2017, 29, 3126.

[23] F. Torricelli , I. Alessandri , E. Macchia , I. Vassalini , M. Maddaloni , L. Torsi , Adv. Mater. Technol. 2022, 7, 2100445.

[24] J. T. Mabeck , J. A. DeFranco , D. A. Bernards , G. G. Malliaras , S. Hocdé , C. J. Chase , Appl. Phys. Lett. 2005, 87, 013503.

[25] G. Frusconi , Z. M. Kovács‐Vajna , P. W. M. Blom , P. Gkoupidenis , F. Torricelli , Adv. Mater. Technol. 2024, 10, 2401440.

[26] L. Huang , D. Zhao , X. Yan , X. Liu , Q. Sun , H. Yang , X. Liu , H. Jia , Adv. Electron. Mater. 2025, 11, 2570007.

[27] R. Granelli , Z. M. Kovács‐Vajna , F. Torricelli , Small 2025, 21, 2410499. 39945058 10.1002/smll.202410499 PMC11922034

[28] A. Makhinia , K. Hübscher , V. Beni , P. A. Ersman , Adv. Mater. Technol. 2022, 7, 61428.

[29] H. Sun , M. Vagin , S. Wang , X. Crispin , R. Forchheimer , M. Berggren , S. Fabiano , Adv. Mater. 2018, 30, 1704916. 10.1002/adma.201704916 29318706

[30] C. Y. Yang , M. A. Stoeckel , T. P. Ruoko , H. Y. Wu , X. Liu , N. B. Kolhe , Z. Wu , Y. Puttisong , C. Musumeci , M. Massetti , H. Sun , K. Xu , D. Tu , W. M. Chen , H. Y. Woo , M. Fahlman , S. A. Jenekhe , M. Berggren , S. Fabiano , Nat. Commun. 2021, 12, 2354. 33883549 10.1038/s41467-021-22528-y PMC8060302

[31] G. Frusconi , Z. M. Kovács‐Vajna , F. Torricelli , Adv. Mater. Technol. 2024, 9, 2400301.

[32] Y. Zhong , Y. Zhang , J. Pu , S. Wustoni , J. Uribe , N. Lopez‐Larrea , A. Marks , I. McCulloch , D. Mecerreyes , D. Baran , S. Inal , Device 2025, 3, 100778.

[33] Y. Zhong , A. Saleh , S. Inal , Macromol. Biosci. 2021, 21, 2100187. 10.1002/mabi.202100187 34463019

[34] P. Romele , P. Gkoupidenis , D. A. Koutsouras , K. Lieberth , Z. M. Kovács‐Vajna , P. W. M. Blom , F. Torricelli , Nat. Commun. 2020, 11, 3743. 32719350 10.1038/s41467-020-17547-0 PMC7385487

[35] S. Wang , L. Han , Y. Zou , B. Liu , Z. He , Y. Huang , Z. Wang , L. Zheng , Y. Hu , Q. Zhao , Y. Sun , Z. Li , P. Gao , X. Chen , X. Guo , L. Li , W. Hu , Sci. Adv. 2023, 9, adj4656. 10.1126/sciadv.adj4656 PMC10699771 38055810

[36] F. Torricelli , L. Colalongo , D. Raiteri , Z. M. Kovács‐Vajna , E. Cantatore , Nat. Commun. 2016, 7, 1. 10.1038/ncomms10550 PMC4740436 26829567

[37] M. Ghittorelli , L. Lingstedt , P. Romele , N. I. Crǎciun , Z. M. Kovács‐Vajna , P. W. M. Blom , F. Torricelli , Nat. Commun. 2018, 9, 1441. 29650956 10.1038/s41467-018-03932-3 PMC5897342

[38] T. Leydecker , Z. M. Wang , F. Torricelli , E. Orgiu , Chem. Soc. Rev. 2020, 49, 7627. 33016288 10.1039/d0cs00106f

[39] K. Lieberth , A. Pavlou , D. Harig , P. W. M. Blom , P. Gkoupidenis , F. Torricelli , Adv. Mater. Technol. 2023, 8, 2201697.

[40] M. Afonso , J. Morgado , L. Alcácer , J. Appl. Phys. 2016, 120, 165502.

[41] E. J. Strand , E. Bihar , S. M. Gleason , S. Han , S. W. Schreiber , M. N. Renny , G. G. Malliaras , R. R. McLeod , G. L. Whiting , Adv. Electron. Mater. 2022, 8, 2100853.

[42] M. Sensi , M. Berto , A. Candini , A. Liscio , A. Cossarizza , V. Beni , F. Biscarini , C. A. Bortolotti , ACSO. 2019, 4, 5374.

[43] N. Fumeaux , C. P. Almeida , S. Demuru , D. Briand , Sci. Rep. 2023, 13, 11467. 37454190 10.1038/s41598-023-38308-1 PMC10349802

[44] S. Demuru , C.‐H. Huang , K. Parvez , R. Worsley , G. Mattana , B. Piro , V. Noël , C. Casiraghi , D. Briand , ACS Appl. Nano Mater. 2022, 5, 1664.

[45] M. Massetti , S. Zhang , P. C. Harikesh , B. Burtscher , C. Diacci , D. T. Simon , X. Liu , M. Fahlman , D. Tu , M. Berggren , S. Fabiano , npj Flexible Electron. 2023, 7, 11.

[46] A. Makhinia , L. Bynens , A. Goossens , J. Deckers , L. Lutsen , K. Vandewal , W. Maes , V. Beni , P. A. Ersman , Adv. Funct. Mater. 2024, 34, 2314857.

[47] R. Granelli , I. Alessandri , P. Gkoupidenis , I. Vassalini , Z. M. Kovács‐Vajna , P. W. M. Blom , F. Torricelli , Small 2022, 18, 2108077. 10.1002/smll.202108077 35642950

[48] C. H. Kim , M. Azimi , J. Fan , H. Nagarajan , M. Wang , F. Cicoira , Nanoscale 2023, 15, 3263. 36722914 10.1039/d2nr06731e

[49] T. N. Mangoma , S. Yamamoto , G. G. Malliaras , R. Daly , Adv. Mater. Technol. 2022, 7, 2000798.

[50] M. Azimi , C. H. Kim , J. Fan , F. Cicoira , Faraday Discuss. 2023, 246, 045006. 10.1039/d3fd00065f 37436097

[51] V. Bertana , G. Scordo , M. Parmeggiani , L. Scaltrito , S. Ferrero , M. G. Gomez , M. Cocuzza , D. Vurro , P. D'Angelo , S. Iannotta , C. F. Pirri , S. L. Marasso , Sci. Rep. 2020, 10, 13335. 32770035 10.1038/s41598-020-70365-8 PMC7414134

[52] J. Fan , C. Montemagno , M. Gupta , Org. Electron. 2019, 73, 122.

[53] D. Majak , J. Fan , M. Gupta , Sens Actuators B Chem 2019, 286, 111.

[54] P. Andersson Ersman , R. Lassnig , J. Strandberg , D. Tu , V. Keshmiri , R. Forchheimer , S. Fabiano , G. Gustafsson , M. Berggren , Nat. Commun. 2019, 10, 5053. 31699999 10.1038/s41467-019-13079-4 PMC6838054

[55] M. Zabihipour , D. Tu , J. Strandberg , M. Berggren , I. Engquist , P. A. Ersman , Adv. Mater. Technol. 2021,34, 2106235.

[56] M. Zabihipour , D. Tu , R. Forchheimer , J. Strandberg , M. Berggren , I. Engquist , P. A. Ersman , Adv. Mater. Technol. 2022, 7, 2106235.

[57] P. C. Hutter , T. Rothlander , G. Scheipl , B. Stadlober , IEEE Trans. Electron Devices 2015, 62,4231.

[58] P. Leleux , J. Rivnay , T. Lonjaret , J. Badier , C. Bénar , T. Hervé , P. Chauvel , G. G. Malliaras , Adv. Healthcare Mater. 2015, 4, 142. 10.1002/adhm.201400356 25262967

[59] X. Wu , T. L. D. Tam , S. Chen , T. Salim , X. Zhao , Z. Zhou , M. Lin , J. Xu , Y. Loo , W. L. Leong , Adv. Mater. 2022, 34, 2206118. 10.1002/adma.202206118 36008368

[60] Y. Xia , W. Zhang , M. Ha , J. H. Cho , M. J. Renn , C. H. Kim , C. D. Frisbie , Adv. Funct. Mater. 2010, 20, 587.

[61] L. Sanchez‐Duenas , E. Gomez , M. Larrañaga , M. Blanco , A. M. Goitandia , E. Aranzabe , J. L. Vilas‐Vilela , Materials 2023, 16, 3940. 37297073 10.3390/ma16113940 PMC10253832

[62] X. Zhou , L. Zhang , Y. Wang , S. Zhao , Y. Zhou , Y. Guo , Y. Wang , J. Liang , H. Chen , Adv. Mater. Technol. 2023, 8, 2201272.

[63] H. Liu , A. Yang , J. Song , N. Wang , P. Lam , Y. Li , H. K. Law , F. Yan , Sci. Adv. 2021, 7, abg8387. 10.1126/sciadv.abg8387 PMC8443172 34524851

[64] P. Lin , F. Yan , J. Yu , H. L. W. Chan , M. Yang , Adv. Mater. 2010, 22, 3655. 20661950 10.1002/adma.201000971

[65] D. A. Koutsouras , M. H. Amiri , P. W. M. Blom , F. Torricelli , K. Asadi , P. Gkoupidenis , Adv. Funct. Mater. 2021, 31, 2011013.

[66] P. Belleri , J. Pons i Tarrés , I. McCulloch , P. W. M. Blom , Z. M. Kovács‐Vajna , P. Gkoupidenis , F. Torricelli , Nat. Commun. 2024, 15, 5350. 38914568 10.1038/s41467-024-49668-1 PMC11196688

[67] T. Sarkar , K. Lieberth , A. Pavlou , T. Frank , V. Mailaender , I. McCulloch , P. W. M. Blom , F. Torricelli , P. Gkoupidenis , Nat. Electron. 2022, 5, 774.

