# Supplementary material for: Organic Electrochemical Transistors Monolithically Integrated with Precise Micro‐Dispensing Enable High‐Performance Biosignals Amplification
Source: Adv Sci (Weinh). 2025 Aug 21;12(40):e08872. doi: 10.1002/advs.202508872 (PMC12561287; doi:10.1002/advs.202508872)
Supplement: Supplementary file 1 — Supporting Information [file ADVS-12-e08872-s002.pdf]

Supporting Information

Organic Electrochemical Transistors Monolithically Integrated with Precise Micro-Dispensing enable High-Performance Biosignals Amplification

*Roberto Granelli, Virginia M. Demartis, Giulia Frusconi, Zsolt M. Kovács-Vajna, and Fabrizio Torricelli**

R. Granelli, V. M. Demartis, G. Frusconi, Zs. M. Kovács-Vajna, F. Torricelli

Department of Information Engineering, University of Brescia, via Branze 38, 25123 Brescia, Italy

E-mail: fabrizio.torricelli@unibs.it

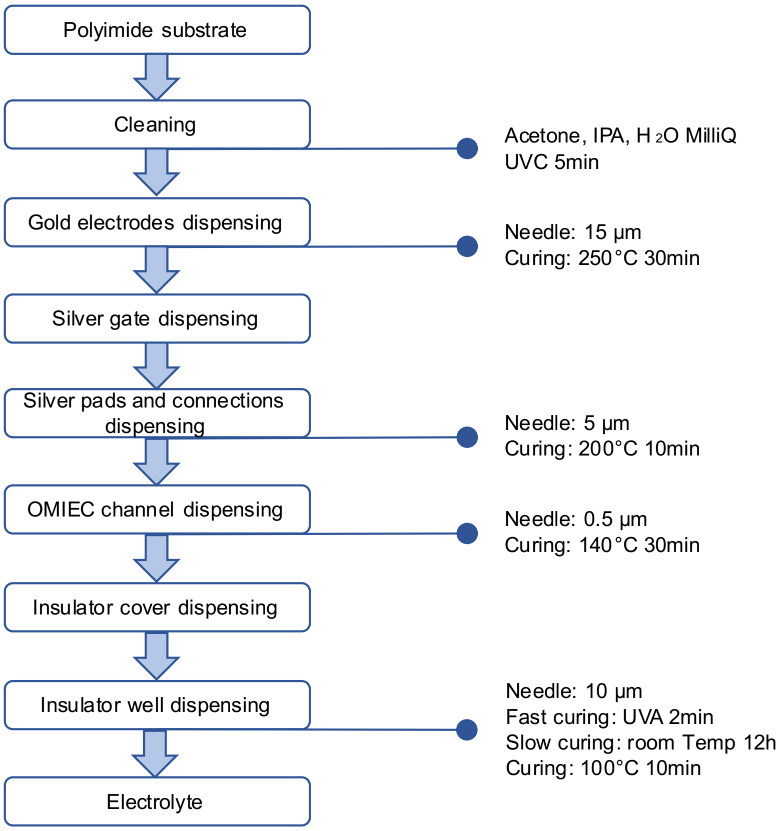

Figure S1. Fabrication process steps of micro-dispensed OECTs. The fabrication process comprises 6 micro-dispensing steps.

Ink	Needle I.D. (μm)	Velocity (mm/s)	Acceleration (mm/s^2)	Printing pressure (mbar)	Non-Printing pressure (mbar)	Delay (ms)
Ag Nanopaste	5	0.1	10	8000	1500	2500
Gold Nanoink	5	1	20	10	10	0
Insulator	5	0.2	10	4500	450	100
PEDOT:PSS	5	1	20	50	50	10

Table S1. Printing parameters used in OECT fabrication. I.D. is the internal diameter.

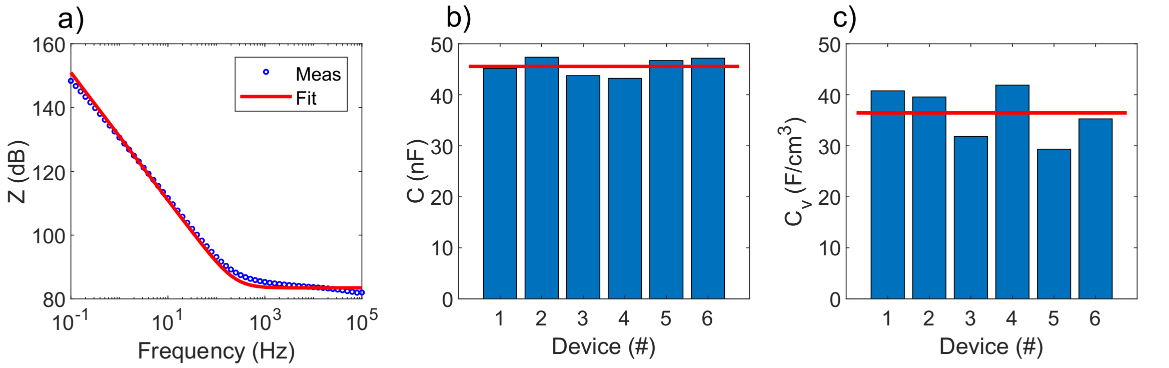

Figure S2. a) Representative measured (symbols) and modelled (line) EIS spectra. We modeled the EIS spectra using the Randles equivalent circuit, composed of a resistor in series with a capacitor, and we found C = 45.2 nF and R = 14.9 kΩ. b) Bar chart of six OECTs channel capacitance. Red line is the mean value C_m = 45.5 nF. c) Corresponding volumetric capacitance (C_V = C/V, where V is the measured volume of the polymeric channel) of six OECTs. Red line is the mean value C_V = 36.45 F/cm^3. EIS measurements were carried out using a DC bias V_{DC} = 0 V and an AC voltage amplitude V_{AC} = 10 mV, in 1 M NaCl aqueous electrolyte.

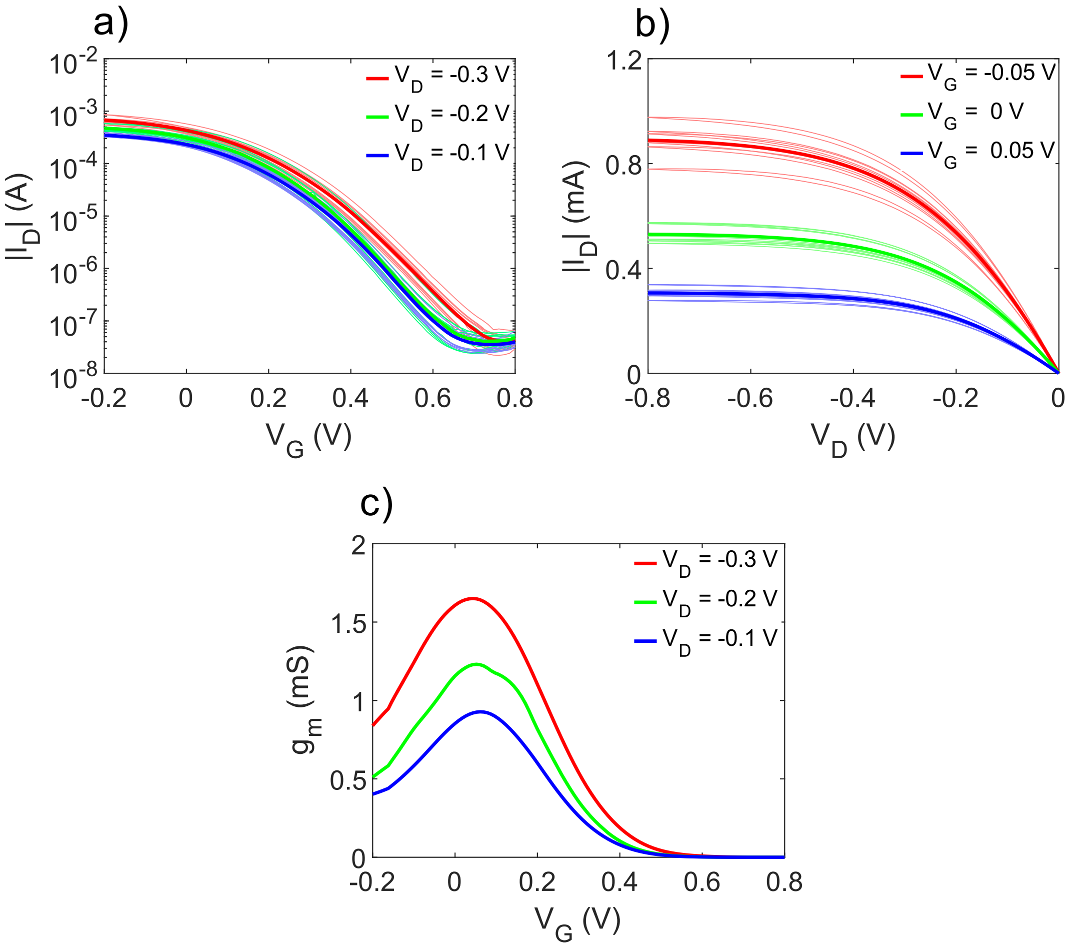

Figure S3. a) Transfer characteristics of six OECTs measured at various drain voltages V_D = –0.3 V (red), –0.2 V (green), and –0.1 V (blue). Thin lines represent individual devices, while thick lines are the average characteristic at each drain voltage. b) Output characteristics of six OECTs measured at gate voltage V_G = –0.05 V (red), 0 V (green), and 0.05 V (blue). Thin lines correspond to individual devices, while thick lines are the average characteristic. c) Corresponding transconductance at various drain voltages. All measurements were performed in 1 M NaCl aqueous electrolyte. OECTs were fabricated on a 50 µm thick polyimide substrate. The channel geometry of the OECTs was as follows: channel width W = 60 µm, channel length L = 12 µm, and average channel thickness t ≈ 750 nm.

Ink	Needle I.D. (μm)	Velocity (mm/s)	Acceleration (mm/s^2)	Printing pressure (mbar)	Non-Printing pressure (mbar)	Delay (ms)
Ag Nanopaste	5	0.4	10	6500	1500	1500
Gold Nanoink	15	1	20	1	1	0
Insulator	10	0.2	10	1500	250	100
PEDOT:PSS	5	1	20	10	10	10

Table S2. Printing parameters used in dot matrices fabrication.

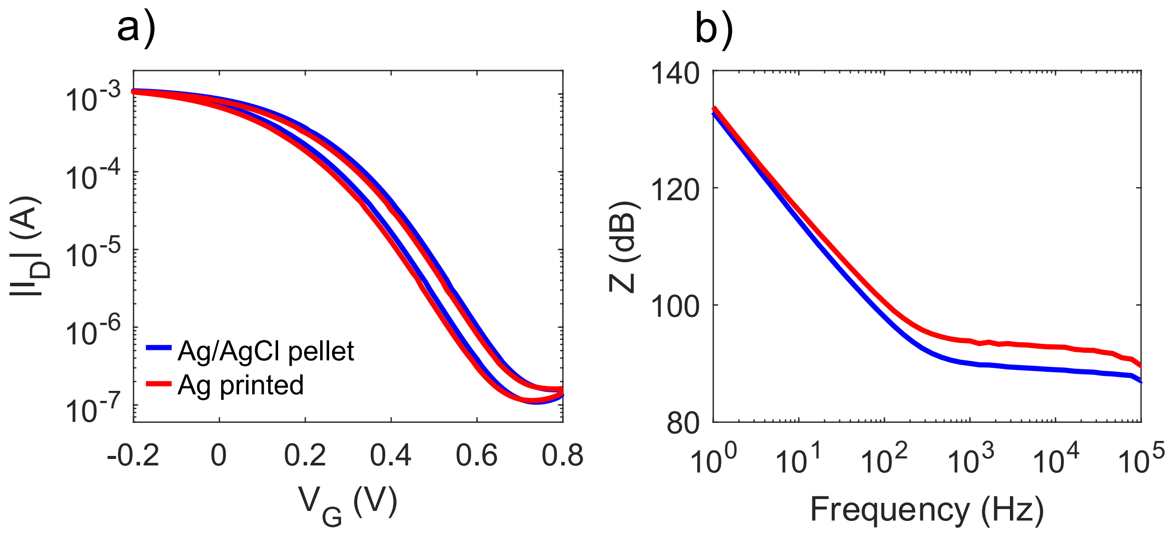

Figure S4. a) Transfer characteristics of an OECT measured at V_D = –0.1 V by using as gate electrode a conventional Ag/AgCl pellet (blue line) and a micro-dispensed silver electrode (red line). b) EIS measurements of an OECT operated in a two-electrode configuration with a Ag/AgCl pellet (blue line) and a micro-dispensed silver electrode (red line). The OECT channel is used as working electrode and the source and drain are connected together. The gate is used as counter and reference electrode. The electrolyte is 0.1 M NaCl solution.

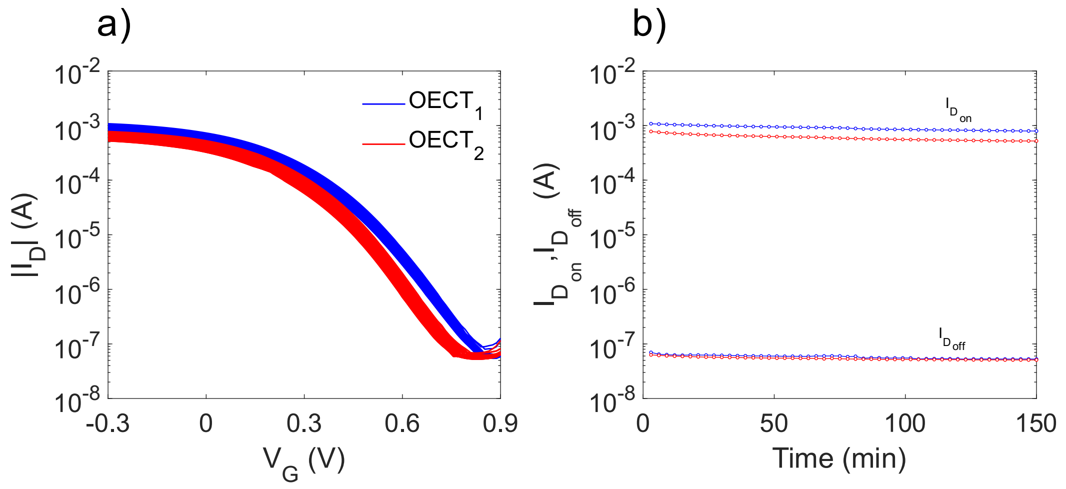

Figure S5. a) Transfer characteristics of two representative OECTs measured at V_D = -0.1 V. All transfer characteristics measured over the subsequent 150 min. period are shown. b) Corresponding on and off drain current over time.

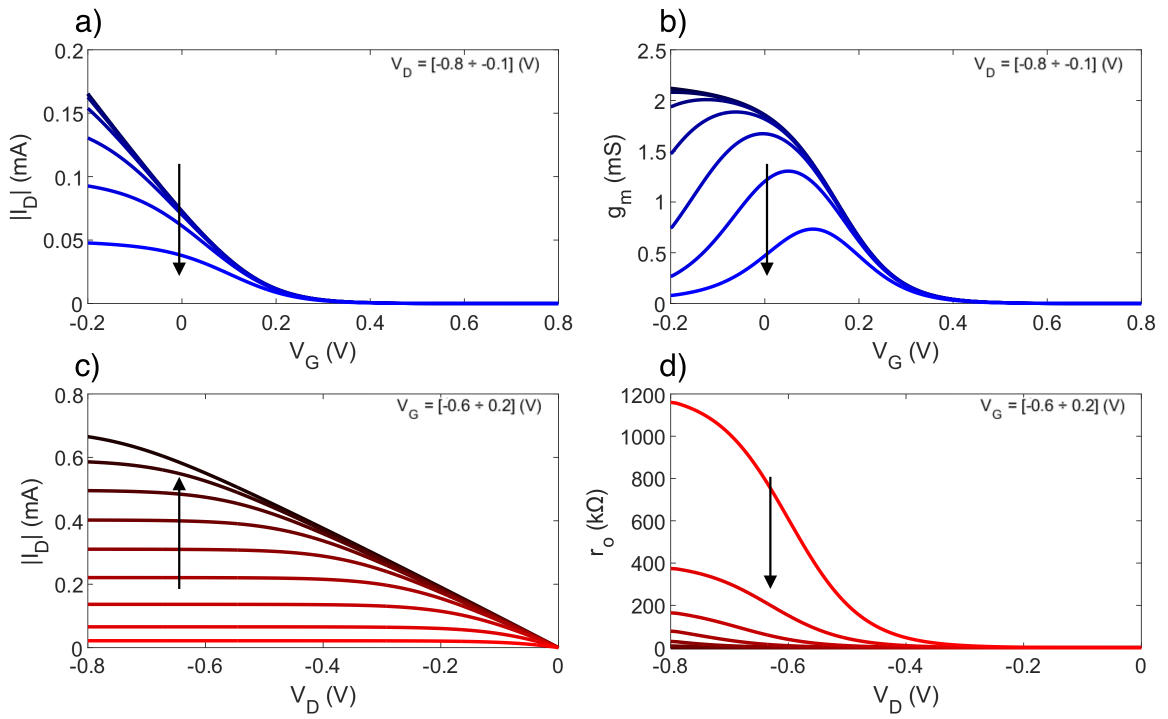

Figure S6. a) Transfer characteristics measured at several drain voltages ($-0.8 \le V_D \le -0.1$ V) and b) corresponding transconductance g_m. c) Output characteristics at several gate voltage ($-0.6 \le V_G \le 0.2$ V) and d) corresponding output resistance r_o. The measurements are performed in 5 M NaCl electrolyte.

Channel material	Gate material	Source/Drain material	Fabrication method	Structure	L (µm)	t (nm)	W (µm)	g_m (mS)	r_o (kΩ)	G_I (V/V)	Ref
PEDOT:PSS	PEDOT:PSS	GO/CNT	Dispensing	Side gate	77		240	7	0.1	0.7	[1]
PEDOT:PSS	Ag/AgCl	Carbon	Dispensing + Direct Write	Side gate	400	100	1000	0.75	3.6	2.7	[2]
P(g42T-T)	Ag/AgCl	Carbon	Screen printing + Spray coating	Side gate	200	20	2000	0.17	352	59.8	[3]
BBL	Ag/AgCl	Carbon	Screen printing + Spray coating	Side gate	200	250	2000	0.18	333	59.9	[3]
pgBTTT	PEDOT:PSS	Carbon	Screen printing + Inkjet printing	Top gate	80	100	375	0.6	50	30	[4]
PEDOT:PSS	Ag/AgCl	Silver	Dispensing	Side gate	1500	1000	1000	1.2	20	24	[5]
PEDOT:PSS	Ag/AgCl	Carbon	3D printing + Inkjet printing	Pellet gate	3000	2100	10000	0.2	15	3	[6]
PEDOT:PSS	Ag/AgCl	Silver	Dispensing	Side gate	118	2000	1000	1.5	2.5	3.8	[7]
PEGDA: PEDOT	Ag/AgCl	PEGDA:PEDOT	Stereolithography	Pellet gate	2000	300	700	2.5	10	25	[8]
PEDOT:PSS	Carbon	Carbon	Screen printing	Side gate	2000	280	3000	0.7	39	27.3	[9]
PEDOT:PSS	Ag/AgCl	Silver	Dispensing	Pellet gate	694	7100	912	30.8	0.8	24.6	[10]
PEDOT:PSS	PEDOT:PSS	Carbon	Screen printing + Inkjet printing	Side gate	3000	500	1000	0.33	2	0.7	[11]
PEDOT:PSS	Graphene	Silver	Inkjet printing	Side gate	3000	1000	1000	0.13	3	0	[12]
PEDOT:PSS	PEDOT:PSS	PEDOT:PSS	Inkjet printing	Side gate	3000		2000	1.67	10	16.7	[13]
PEDOT:PSS	Ag/AgCl	Silver	Inkjet printing	Side gate	700	2500	700	5	2	10	[14]
PEDOT:PSS	Silver	Silver	Dispensing	Side gate	417	9000	953	34	3.8	129.2	[15]
PEDOT:PSS	Silver	Gold	Micro-dispensing	Side gate	12	750	60	2	165	330	This work

Table S3. Comparison of OECTs fabricated with fully-additive manufacturing methods.

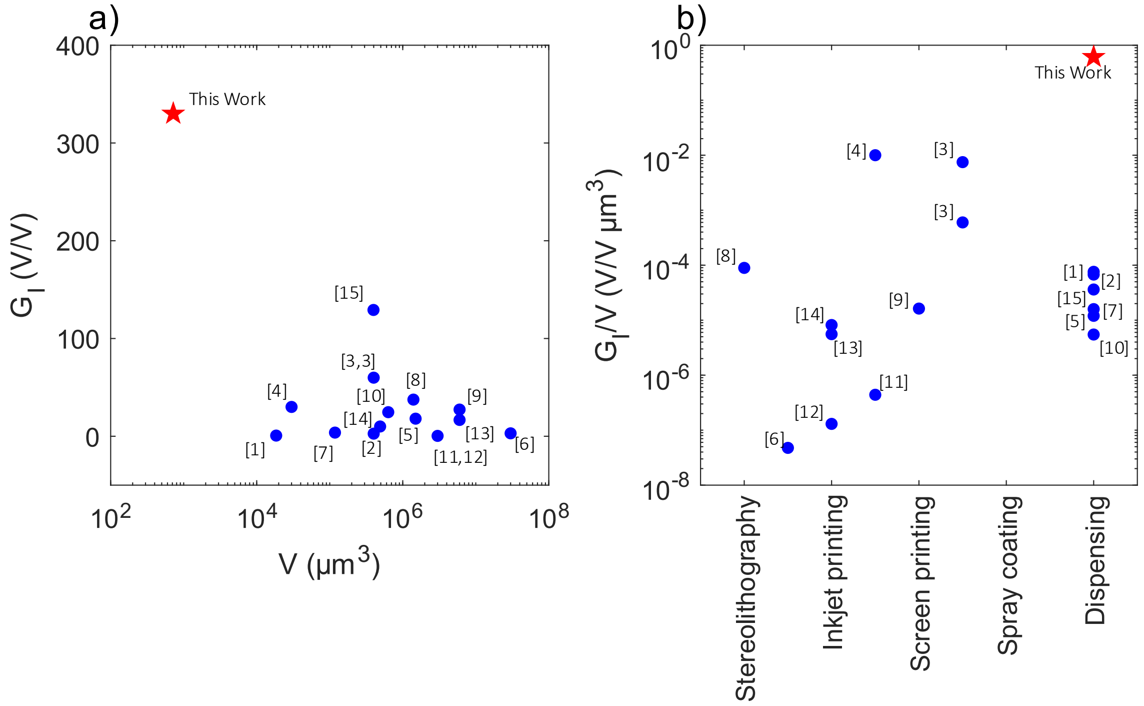

Figure S7. Gain benchmarking. a) Comparison of the gain of the fully printed OECTs versus the channel area. b) Comparison of the gain normalized to the channel volume of the OECTs considering various OECT fabrication methods.

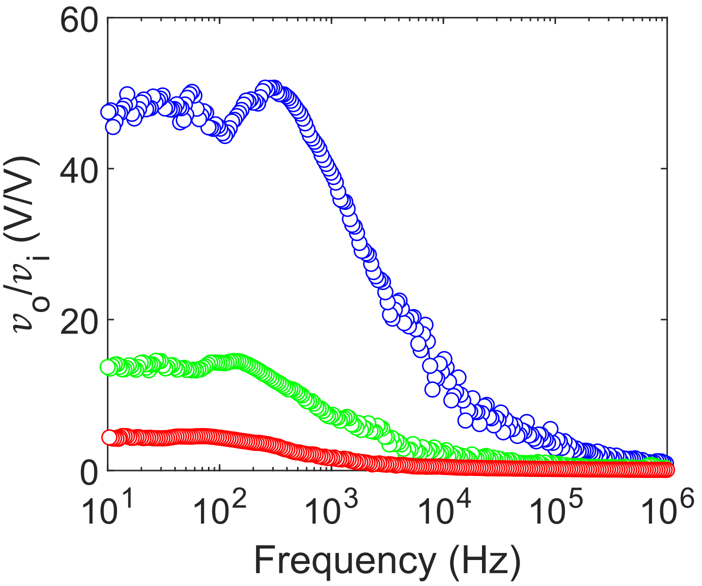

Figure S8. Voltage gain of the unipolar OECT amplifier, using a NaCl aqueous electrolyte with ion concentration of 5 M (blue), 1 M (green), and 0.1 M (red).

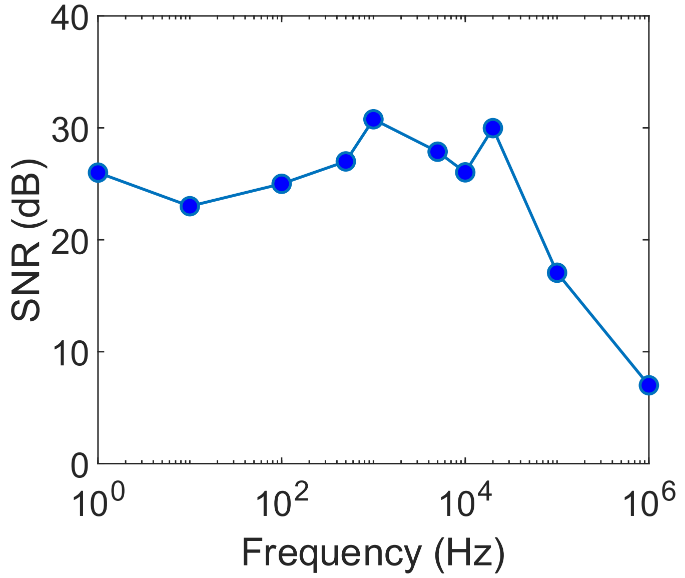

Figure S9. Signal to noise ratio measured at various frequencies with NaCl electrolyte with 5 M ion concentration.

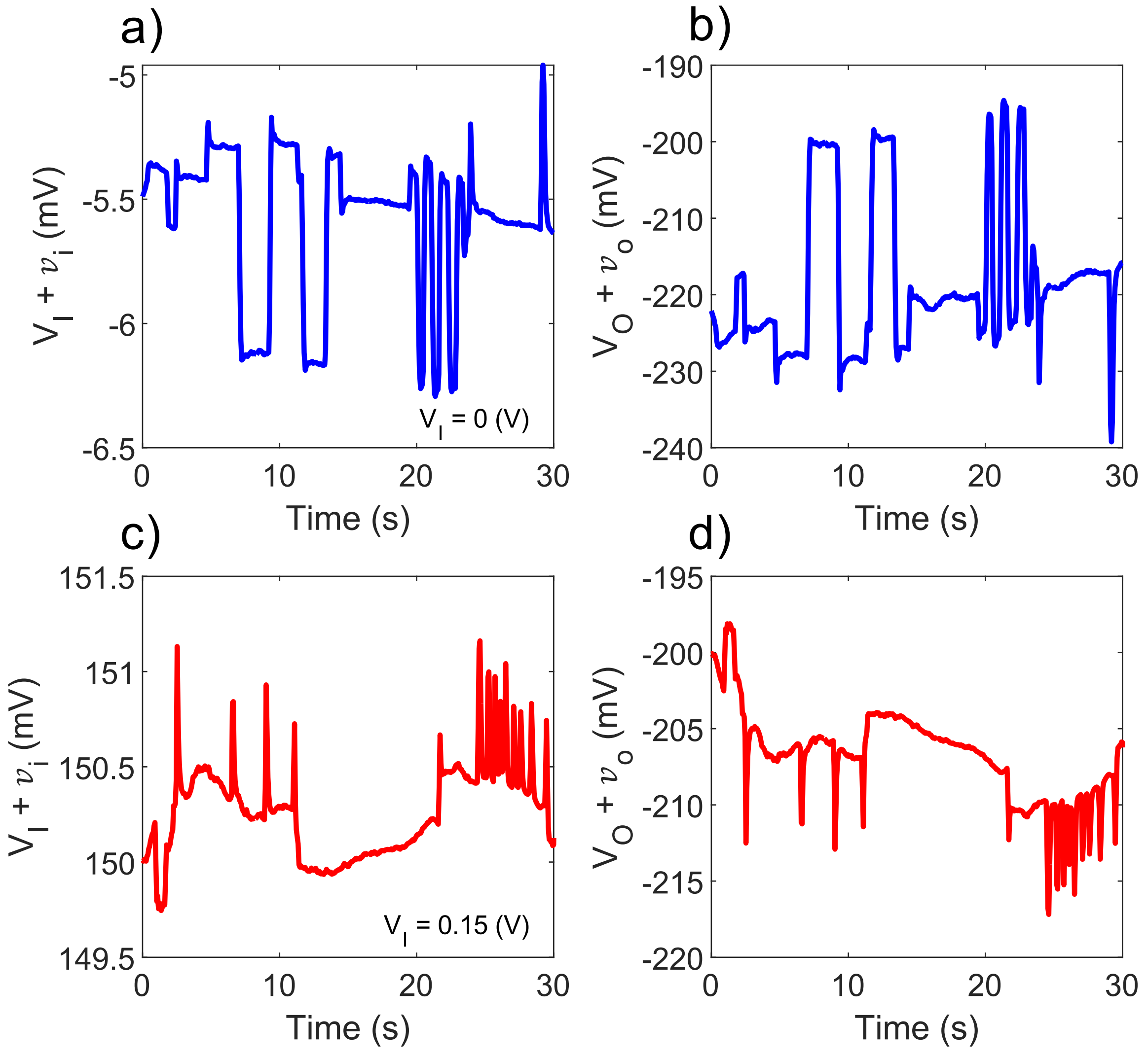

Figure S10. a) Input and b) output voltage of the OECT-based amplifier used for electrooculography. DC input voltage V_I = 0 V, and electrolyte is 5 M NaCl. d) Input and e) output voltage of the OECT-based amplifier used for electrooculography. DC input voltage V_I = 0.15 V, and electrolyte is 1 M NaCl.

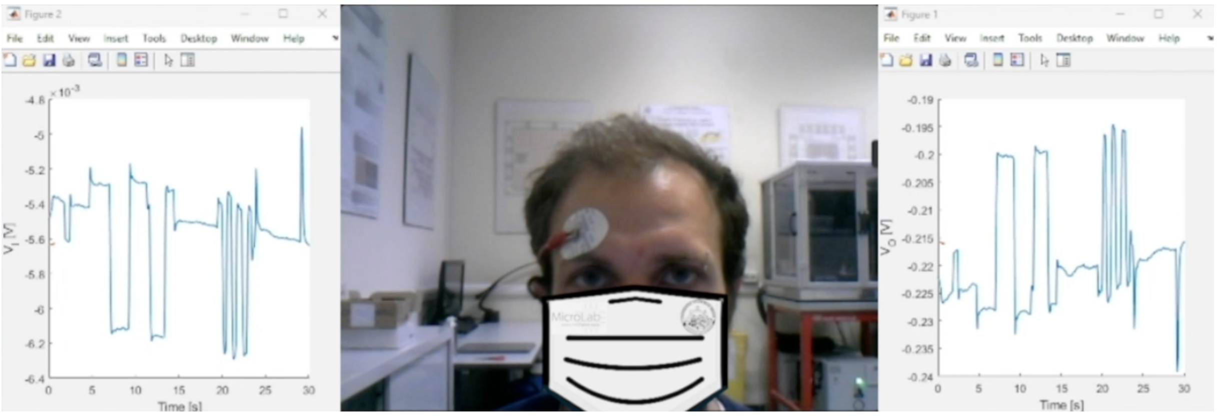

Movie S1. Experimental set-up during EOG measurements. Real time measurements and amplification of the EOG signal.

References

[1] M. Massetti, S. Zhang, P. C. Harikesh, B. Burtscher, C. Diacci, D. T. Simon, X. Liu, M. Fahlman, D. Tu, M. Berggren, S. Fabiano, *npj Flexible Electronics* **2023**, *7*, DOI 10.1038/s41528-023-00245-4.

[2] R. Granelli, I. Alessandri, P. Gkoupidenis, I. Vassalini, Z. M. Kovács-Vajna, P. W. M. Blom, F. Torricelli, *Small* **2022**, *18*, DOI 10.1002/smll.202108077.

[3] C. Y. Yang, D. Tu, T. P. Ruoko, J. Y. Gerasimov, H. Y. Wu, P. C. Harikesh, M. Massetti, M. A. Stoeckel, R. Kroon, C. Müller, M. Berggren, S. Fabiano, *Adv Electron Mater* **2022**, *8*, DOI 10.1002/aelm.202100907.

[4] A. Makhinia, L. Bynens, A. Goossens, J. Deckers, L. Lutsen, K. Vandewal, W. Maes, V. Beni, P. Andersson Ersman, *Adv Funct Mater* **2024**, *34*, DOI 10.1002/adfm.202314857.

[5] C. H. Kim, M. Azimi, J. Fan, H. Nagarajan, M. Wang, F. Cicoira, *Nanoscale* **2023**, 15, DOI 10.1039/D2NR06731E.

[6] T. N. Mangoma, S. Yamamoto, G. G. Malliaras, R. Daly, *Adv Mater Technol* **2022**, *7*, DOI 10.1002/admt.202000798.

[7] M. Azimi, C. H. Kim, J. Fan, F. Cicoira, *Faraday Discuss* **2023**, 246, DOI 10.1039/D3FD00065F.

[8] V. Bertana, G. Scordo, M. Parmeggiani, L. Scaltrito, S. Ferrero, M. G. Gomez, M. Cocuzza, D. Vurro, P. D'Angelo, S. Iannotta, C. F. Pirri, S. L. Marasso, *Sci Rep* **2020**, *10*, DOI 10.1038/s41598-020-70365-8.

[9] M. Sensi, M. Berto, A. Candini, A. Liscio, A. Cossarizza, V. Beni, F. Biscarini, C. A. Bortolotti, *ACS Omega* **2019**, *4*, DOI 10.1021/acsomega.8b03319.

[10] J. Fan, C. Montemagno, M. Gupta, *Org Electron* **2019**, 73, DOI 10.1016/j.orgel.2019.06.012

[11] N. Fumeaux, C. P. Almeida, S. Demuru, D. Briand, *Sci Rep* **2023**, *13*, DOI 10.1038/s41598-023-38308-1.

[12] S. Demuru, C.-H. Huang, K. Parvez, R. Worsley, G. Mattana, B. Piro, V. Noël, C. Casiraghi, D. Briand, *ACS Appl Nano Mater* **2022**, *5*, DOI 10.1021/acsanm.1c04434.

[13] M. Afonso, J. Morgado, L. Alcácer, *J Appl Phys* **2016**, *120*, DOI 10.1063/1.4966651.

[14] E. J. Strand, E. Bihar, S. M. Gleason, S. Han, S. W. Schreiber, M. N. Renny, G. G. Malliaras, R. R. McLeod, G. L. Whiting, *Adv Electron Mater* **2022**, *8*, DOI 10.1002/aelm.202100853.

[15] D. Majak, J. Fan, M. Gupta, *Sens Actuators B Chem* **2019**, DOI 10.1016/j.snb.2019.01.120.
